# Pharmacological and electronic cigarette interventions for smoking cessation in adults: component network meta‐analyses

**DOI:** 10.1002/14651858.CD015226.pub2

**Published:** 2023-09-12

**Authors:** Nicola Lindson, Annika Theodoulou, José M Ordóñez-Mena, Thomas R Fanshawe, Alex J Sutton, Jonathan Livingstone-Banks, Anisa Hajizadeh, Sufen Zhu, Paul Aveyard, Suzanne C Freeman, Sanjay Agrawal, Jamie Hartmann-Boyce

**Affiliations:** Nuffield Department of Primary Care Health SciencesUniversity of OxfordOxfordUK; Department of Health SciencesUniversity of LeicesterLeicesterUK; Department of Respiratory SciencesUniversity of LeicesterLeicesterUK

**Keywords:** Adult, Female, Humans, Pregnancy, Bupropion, Bupropion/therapeutic use, Electronic Nicotine Delivery Systems, Nicotine, Nicotine/adverse effects, Nortriptyline, Nortriptyline/therapeutic use, Smoking Cessation, Varenicline, Varenicline/therapeutic use

## Abstract

**Background:**

Tobacco smoking is the leading preventable cause of death and disease worldwide. Stopping smoking can reduce this harm and many people would like to stop. There are a number of medicines licenced to help people quit globally, and e‐cigarettes are used for this purpose in many countries. Typically treatments work by reducing cravings to smoke, thus aiding initial abstinence and preventing relapse. More information on comparative effects of these treatments is needed to inform treatment decisions and policies.

**Objectives:**

To investigate the comparative benefits, harms and tolerability of different smoking cessation pharmacotherapies and e‐cigarettes, when used to help people stop smoking tobacco.

**Search methods:**

We identified studies from recent updates of Cochrane Reviews investigating our interventions of interest. We updated the searches for each review using the Cochrane Tobacco Addiction Group (TAG) specialised register to 29 April 2022.

**Selection criteria:**

We included randomised controlled trials (RCTs), cluster‐RCTs and factorial RCTs, which measured smoking cessation at six months or longer, recruited adults who smoked combustible cigarettes at enrolment (excluding pregnant people) and randomised them to approved pharmacotherapies and technologies used for smoking cessation worldwide (varenicline, cytisine, nortriptyline, bupropion, nicotine replacement therapy (NRT) and e‐cigarettes) versus no pharmacological intervention, placebo (control) or another approved pharmacotherapy. Studies providing co‐interventions (e.g. behavioural support) were eligible if the co‐intervention was provided equally to study arms.

**Data collection and analysis:**

We followed standard Cochrane methods for screening, data extraction and risk of bias (RoB) assessment (using the RoB 1 tool). Primary outcome measures were smoking cessation at six months or longer, and the number of people reporting serious adverse events (SAEs). We also measured withdrawals due to treatment. We used Bayesian component network meta‐analyses (cNMA) to examine intervention type, delivery mode, dose, duration, timing in relation to quit day and tapering of nicotine dose, using odds ratios (OR) and 95% credibility intervals (CrIs). We calculated an effect estimate for combination NRT using an additive model. We evaluated the influence of population and study characteristics, provision of behavioural support and control arm rates using meta‐regression. We evaluated certainty using GRADE.

**Main results:**

Of our 332 eligible RCTs, 319 (835 study arms, 157,179 participants) provided sufficient data to be included in our cNMA. Of these, we judged 51 to be at low risk of bias overall, 104 at high risk and 164 at unclear risk, and 118 reported pharmaceutical or e‐cigarette/tobacco industry funding. Removing studies at high risk of bias did not change our interpretation of the results.

**Benefits**

We found high‐certainty evidence that nicotine e‐cigarettes (OR 2.37, 95% CrI 1.73 to 3.24; 16 RCTs, 3828 participants), varenicline (OR 2.33, 95% CrI 2.02 to 2.68; 67 RCTs, 16,430 participants) and cytisine (OR 2.21, 95% CrI 1.66 to 2.97; 7 RCTs, 3848 participants) were associated with higher quit rates than control. In absolute terms, this might lead to an additional eight (95% CrI 4 to 13), eight (95% CrI 6 to 10) and seven additional quitters per 100 (95% CrI 4 to 12), respectively. These interventions appeared to be more effective than the other interventions apart from combination NRT (patch and a fast‐acting form of NRT), which had a lower point estimate (calculated additive effect) but overlapping 95% CrIs (OR 1.93, 95% CrI 1.61 to 2.34). There was also high‐certainty evidence that nicotine patch alone (OR 1.37, 95% CrI 1.20 to 1.56; 105 RCTs, 37,319 participants), fast‐acting NRT alone (OR 1.41, 95% CrI 1.29 to 1.55; 120 RCTs, 31,756 participants) and bupropion (OR 1.43, 95% CrI 1.26 to 1.62; 71 RCTs, 14,759 participants) were more effective than control, resulting in two (95% CrI 1 to 3), three (95% CrI 2 to 3) and three (95% CrI 2 to 4) additional quitters per 100 respectively.

Nortriptyline is probably associated with higher quit rates than control (OR 1.35, 95% CrI 1.02 to 1.81; 10 RCTs, 1290 participants; moderate‐certainty evidence), resulting in two (CrI 0 to 5) additional quitters per 100. Non‐nicotine/placebo e‐cigarettes (OR 1.16, 95% CrI 0.74 to 1.80; 8 RCTs, 1094 participants; low‐certainty evidence), equating to one additional quitter (95% CrI ‐2 to 5), had point estimates favouring the intervention over control, but CrIs encompassed the potential for no difference and harm. There was low‐certainty evidence that tapering the dose of NRT prior to stopping treatment may improve effectiveness; however, 95% CrIs also incorporated the null (OR 1.14, 95% CrI 1.00 to 1.29; 111 RCTs, 33,156 participants). This might lead to an additional one quitter per 100 (95% CrI 0 to 2).

**Harms**

There were insufficient data to include nortriptyline and non‐nicotine EC in the final SAE model. Overall rates of SAEs for the remaining treatments were low (average 3%). Low‐certainty evidence did not show a clear difference in the number of people reporting SAEs for nicotine e‐cigarettes, varenicline, cytisine or NRT when compared to no pharmacotherapy/e‐cigarettes or placebo. Bupropion may slightly increase rates of SAEs, although the CrI also incorporated no difference (moderate certainty). In absolute terms bupropion may cause one more person in 100 to experience an SAE (95% CrI 0 to 2).

**Authors' conclusions:**

The most effective interventions were nicotine e‐cigarettes, varenicline and cytisine (all high certainty), as well as combination NRT (additive effect, certainty not rated). There was also high‐certainty evidence for the effectiveness of nicotine patch, fast‐acting NRT and bupropion. Less certain evidence of benefit was present for nortriptyline (moderate certainty), non‐nicotine e‐cigarettes and tapering of nicotine dose (both low certainty).

There was moderate‐certainty evidence that bupropion may slightly increase the frequency of SAEs, although there was also the possibility of no increased risk. There was no clear evidence that any other tested interventions increased SAEs. Overall, SAE data were sparse with very low numbers of SAEs, and so further evidence may change our interpretation and certainty.

Future studies should report SAEs to strengthen certainty in this outcome. More head‐to‐head comparisons of the most effective interventions are needed, as are tests of combinations of these. Future work should unify data from behavioural and pharmacological interventions to inform approaches to combined support for smoking cessation.

## Plain language summary

**How effective are medications and e‐cigarettes for quitting smoking, and what works best?**

**Key messages**

Some medicines and e‐cigarettes (handheld devices that work by heating liquid that usually contains nicotine and flavourings) can help people to quit smoking for six months or longer.

E‐cigarettes, and the medicines cytisine (otherwise known as Tabex) and varenicline (otherwise known as Chantix and Champix), appear to help the most people to quit smoking, followed by using two types of nicotine replacement therapy at once (nicotine patch and another type, such as gum or lozenge).

We need more evidence on possible long‐term harms of e‐cigarettes and medicines to help people quit smoking, but there were very low numbers of serious harms found.

**Stopping smoking**

Tobacco smoking is bad for people's health and stopping can lead to significant improvements. Most people would like to quit smoking and there are medicines and e‐cigarettes available to help people to do that. These medicines are called nicotine replacement therapy, cytisine, varenicline, bupropion (sometimes known as Zyban or Wellbutrin) and nortriptyline (sometimes known as Norpress). Nortriptyline is only available for quitting smoking in New Zealand, and cytisine is not available in many countries. At time of writing, there are shortages of varenicline due to a manufacturing issue. These medicines and e‐cigarettes can be provided alongside behavioural support, such as counselling. Stop smoking medications and e‐cigarettes are designed to reduce people's craving to smoke. By finding out more information on how these treatments compare to each other, we hope this review will be used to decide the best treatments to help people stop smoking.

**Why we did this Cochrane Review**

We wanted to find out:

‐ which treatments (medicines and e‐cigarettes) help people to stop smoking;

‐ how these treatments compare to each other;

‐ whether there are ways of providing these treatments that mean they are more likely to help people stop smoking (e.g. different doses or treatment lengths);

‐ whether these treatments are likely to cause serious harms; and

‐ whether certain treatments are better tolerated, as indicated by fewer people leaving a study due to treatment.

**What did we do?**

We searched for studies that looked at these treatments to help adults quit smoking. We looked for randomised controlled trials, where the treatments people received were decided at random. This type of study usually gives the most reliable evidence about the effects of treatments. We compared all treatments with each other using a method called network meta‐analysis.

**Search date:** 29 April 2022

**What we found**

We found 332 studies that met our criteria and 319 of these provided information that we could use in our analyses. These included 157,179 adults who smoked tobacco cigarettes. Most of the studies took place in the USA or Europe. The studies compared the effects of the stop smoking treatments listed above with:

‐ no medicine/e‐cigarettes for stopping smoking;

‐ e‐cigarettes that did not contain nicotine;

‐ placebo (a dummy medication); and

‐ other types of stop smoking medicine or e‐cigarettes.

**What are the main results of our review?**

E‐cigarettes, varenicline and cytisine were most likely to help people quit smoking. For every 100 people, 10 to 19 are likely to quit using an e‐cigarette; 12 to 16 using varenicline; and 10 to 18 using cytisine. This is compared to the 6 in 100 people likely to quit when using no medicine/e‐cigarette or placebo. People using two forms of nicotine replacement therapy at the same time, for example, a combination of nicotine patch and nicotine gum, seemed to have similar rates of quitting to people using e‐cigarettes, varenicline and cytisine. Nicotine patches alone, another form of nicotine replacement therapy alone (such as gum, lozenge) and bupropion appeared to help fewer people quit but still work better than no medicine/e‐cigarette or placebo (8, 9 and 9 people per 100, respectively). Nortriptyline appeared to result in the lowest number of people quitting smoking; for every 100 people using nortriptyline 6 to 11 are likely to quit.

We are moderately confident that bupropion could rarely cause some serious health effects. The information we have for other treatments does not provide clear evidence of serious harms. For all treatments, findings suggest very few people experience serious harms when using them.

**How confident are we in our results?**

We are confident that e‐cigarettes, cytisine, varenicline, nicotine replacement therapy and bupropion help people stop smoking. We do not expect more evidence will change these results. However, more evidence on how these treatments compare to one another, particularly in relation to harms, would be useful. Due to the nature of our analyses we were not able to judge our confidence in the evidence for combination nicotine replacement therapy (two types used together). We are moderately confident that nortriptyline also helps people to stop smoking, but are less confident in our results for non‐nicotine e‐cigarettes and for potential harms of most of the treatments. We still need more evidence on potential harms and hope more studies will report on these in future; however, nicotine replacement therapy has been used since the 1980s with no evidence of serious harms.

## Summary of findings

**Summary of findings 1:** Summary of findings table: components of pharmacological and e‐cigarette interventions for smoking cessation: smoking cessation at 6+ months

**Components of pharmacological and e‐cigarette interventions for smoking cessation: smoking cessation at 6 + months**							
**Population:** adults (aged ≥ 18 years) who smoked cigarettes **Components:** components of pharmacological and e‐cigarette (EC) interventions for smoking cessation **Comparator:** no pharmacological or e‐cigarette intervention (64 RCTs of 15,793 participants had data on this component) **Outcome:** smoking cessation at 6 months to 5 years (although predominantly 6 months to 12 months) **Setting:** predominantly USA and Europe							
**Component**	**Number of participants (studies) with data on component**	Relative effect* (95% CrI)	Anticipated absolute effect**			**Certainty of the evidence**	**Notes**
			**Without intervention**	**With intervention**	**Difference**		
**Varenicline**	16,430 (67 RCTs)	**OR 2.33** (2.02 to 2.68)	6 per 100	14 per 100 (12 to 16)	8 per 100 (6 to 10)	**High**^a^	Prediction interval: 1.31 to 4.11
**Cytisine**	3848 (7 RCTs)	**OR 2.21** (1.66 to 2.97)	6 per 100	13 per 100 (10 to 18)	7 per 100 (4 to 12)	**High**	Prediction interval: 1.19 to 4.22
**Nicotine patch**	37,319 (105 RCTs)	**OR 1.37** (1.20 to 1.56)	6 per 100	8 per 100 (7 to 9)	2 per 100 (1 to 3)	**High**^b^	Prediction interval: 0.77 to 2.41
**Fast‐acting NRT** **(nicotine other)**	31,756 (120 RCTs)	**OR 1.41** (1.29 to 1.55)	6 per 100	9 per 100 (8 to 9)	3 per 100 (2 to 3)	**High**^b^	Prediction interval: 0.81 to 2.49
**Nicotine EC**	3828 (16 RCTs)	**OR 2.37** (1.73 to 3.24)	6 per 100	14 per 100 (10 to 19)	8 per 100 (4 to 13)	**High**	Prediction interval: 1.26 to 4.48
**Non‐nicotine/placebo EC**	1094 (8 RCTs)	**OR 1.16** (0.74 to 1.80)	6 per 100	7 per 100 (4 to 11)	1 per 100 (–2 to 5)	**Low**^c^	Prediction interval: 0.57 to 2.36
**Bupropion**	14,759 (71 RCTs)	**OR 1.43** (1.26 to 1.62)	6 per 100	9 per 100 (8 to 10)	3 per 100 (2 to 4)	**High**^a,b^	Prediction interval: 0.81 to 2.52
**Nortriptyline**	1290 (10 RCTs)	**OR 1.35** (1.02 to 1.81)	6 per 100	8 per 100 (6 to 11)	2 per 100 (0 to 5)	**Moderate**^b,d^	Prediction interval: 0.72 to 2.55
**Nicotine tapering**	33,156 (111 RCTs)	**OR 1.14** (1.00 to 1.29)	6 per 100	7 per 100 (6 to 8)	1 per 100 (0 to 2)	**Low**^d,e^	Prediction interval: 0.64 to 2.00
**Combination nicotine replacement therapy**: this is not included as a row in the summary of findings table as it was not a single component in our analyses. As it is commonly used in practice, we calculated an effect estimate for it (additive on the log scale, assuming no interaction): **OR 1.93, 95% CrI 1.61 to 2.34**							
Network meta‐analysis summary of findings table definitions *Estimates are reported as OR. Results are expressed in CrIs as opposed to CIs as a Bayesian analysis has been conducted. **Anticipated absolute effect compared two risks by calculating the difference between the risks of the intervention component with the risk of the minimal intervention comparator (assumed to be 60 per 1000 based on mean quit rate in minimal intervention arms in Hartmann‐Boyce 2022a). **CI:** confidence interval; **CrI:** credibility interval; **EC:** e‐cigarette; **NRT:** nicotine replacement therapy; **OR:** odds ratio; **RCT:** randomised controlled trial.							
**GRADE Working Group grades of evidence** **High certainty:** we are very confident that the true effect lies close to that of the estimate of effect. **Moderate certainty:** we are moderately confident in the effect estimate: the true effect is likely to be close to the estimate of the effect, but there is a possibility that it is substantially different. **Low certainty:** our confidence in the effect estimate is limited: the true effect may be substantially different from the estimate of the effect. **Very low certainty:** we have very little confidence in the effect estimate: the true effect is likely to be substantially different from the estimate of the effect.							

^a^Funnel plot showed some asymmetry; however, additional small studies favouring control would not be expected to change the interpretation of results. ^b^Did not downgrade due to inconsistency despite prediction intervals encompassing no difference/clinically meaningful harm; this is because pairwise meta‐analyses for these comparisons show low levels of statistical heterogeneity (I^2^ < 25%) and component effect estimates are consistent with those from pairwise meta‐analyses. This suggests that the indirect evidence is what is introducing the inconsistency. Indirect evidence does not change the magnitude or direction of effect. ^c^Downgraded two levels due to imprecision: CrIs encompassed clinically significant benefit as well as clinically significant harm. ^d^Downgraded one level due to imprecision: CrIs encompassed clinically significant benefit as well as no clinically significant difference. ^e^Downgraded one level due to inconsistency; prediction intervals encompass clinically significant harm and clinically significant benefit. Only two studies contribute to direct comparison in pairwise meta‐analysis.

**Summary of findings 2:** Summary of findings table: components of pharmacological and e‐cigarette interventions for smoking cessation: serious adverse events

**Components of pharmacological and e‐cigarette interventions for smoking cessation: serious adverse events (SAEs)**							
**Population:** adults (aged ≥ 18 years) who smoked cigarettes **Components:** components of pharmacological and e‐cigarette (EC) interventions for smoking cessation **Comparator:** placebo e‐cigarette (EC), placebo and no pharmacotherapy (64 RCTs of 15,793 participants had data on these components) **Outcome:** serious adverse events measured at various time points (time point often unreported) **Setting:** predominantly USA and Europe							
**Component**	**Number of participants (studies) with data on component**	Relative effect* (95% CrI)	Anticipated absolute effect**			**Certainty of the evidence**	**Notes**
			**Without intervention**	**With intervention**	**Difference**		
**Varenicline**	13,407 (42 RCTs)	**OR 1.18** (0.93 to 1.49)	3 per 100	3 per 100 (2 to 4)	0 per 100 (‐1 to 1)	**Low**^a^	In the model including control arm SAE as a covariate, the point estimate increases and the CrI no longer incorporates the null (OR 1.67, 95% CrI 1.24 to 2.18) Prediction interval: 0.51 to 2.71
**Cytisine**	2915 (5 RCTs)	**OR 0.94** (0.58 to 1.50)	3 per 1000	2 per 100 (1 to 4)	‐1 per 100 (‐2 to 1)	**Low**^a^	Prediction interval: 0.37 to 2.40
**Nicotine patch**	12,602 (27 RCTs)	**OR 0.96** (0.71 to 1.29)	3 per 100	3 per 100 (2 to 3)	0 per 100 (‐1 to 0)	**Low**^a^	Prediction interval: 0.41 to 2.26
**Fast‐acting NRT (nicotine other)**	5551 (18 RCTs)	**OR 1.07** (0.75 to 1.54)	3 per 100	3 per 100 (2 to 4)	0 per 100 (‐1 to 1)	**Low**^a^	Prediction interval: 0.45 to 2.64
**Nicotine EC**	1642 (7 RCTs)	**OR 0.79** (0.50 to 1.23)	3 per 100	2 per 100 (1 to 3)	‐1 per 100 (‐2 to 0)	**Low**^a^	Prediction interval: 0.31 to 1.96
**Non‐nicotine/placebo EC**	n/a	n/a	n/a	n/a	n/a	n/a	There was insufficient evidence on SAEs to calculate an effect estimate Prediction interval: n/a
**Bupropion**	7231 (22 RCTs)	**OR 1.35** (0.97 to 1.92)	3 per 100	4 per 100 (3 to 5)	1 per 100 (0 to 2)	**Moderate**^b,c^	In the model including control arm SAE as a covariate, the point estimate increases and the CrI no longer incorporates the null (OR 1.44, 95% CrI 1.02 to 2.01) Prediction interval: 0.58 to 3.28
**Nortriptyline**	n/a	n/a	n/a	n/a	n/a	n/a	There was insufficient evidence on SAEs in nortriptyline studies to calculate an effect estimate Prediction interval: n/a
**Combination nicotine replacement therapy**: this is not included as a row in the summary of findings table as it was not a single component in our analyses. As it is commonly used in practice, we calculated an effect estimate for it (additive on the log scale, assuming no interaction): **OR 1.03, 95% CrI 0.68 to 1.56**							
Network meta‐analysis summary of findings table definitions *Estimates are reported as OR. Results are expressed in CrIs as opposed to CIs as a Bayesian analysis has been conducted. **Anticipated absolute effect compared two risks by calculating the difference between the risks of the intervention component with the risk of the minimal intervention comparator (assumed to be 26 per 1000 based on mean SAE rate in the placebo arms). **CIs:** confidence intervals; **CrI:** credibility interval; **EC:** e‐cigarette; **NRT:** nicotine replacement therapy; **OR:** odds ratio; **RCT:** randomised controlled trial; **SAE:** serious adverse events.							
**GRADE Working Group grades of evidence** **High certainty:** we are very confident that the true effect lies close to that of the estimate of effect. **Moderate certainty:** we are moderately confident in the effect estimate: the true effect is likely to be close to the estimate of the effect, but there is a possibility that it is substantially different. **Low certainty:** our confidence in the effect estimate is limited: the true effect may be substantially different from the estimate of the effect. **Very low certainty:** we have very little confidence in the effect estimate: the true effect is likely to be substantially different from the estimate of the effect.							

^a^Downgraded two levels due to imprecision: CrIs encompassed clinically significant benefit as well as clinically significant harm. ^b^Downgraded one level due to imprecision: CrIs encompassed no difference as well as clinically significant harm. ^c^Did not downgrade due to inconsistency despite prediction intervals encompassing no difference/clinically meaningful harm; this is because pairwise meta‐analysis for this comparison shows low levels of statistical heterogeneity (I^2^ < 25%) and component effect estimates are consistent with those from pairwise meta‐analysis. This suggests that the indirect evidence is what is introducing the inconsistency. Indirect evidence does not change the interpretation of effect.

## Background

### Description of the condition

Globally, tobacco smoking is a leading cause of preventable death and disease (WHO 2021a). It is also a key driver of health inequalities, disproportionately affecting vulnerable populations; for example, people with low incomes and mental health conditions (ASH 2019). However, cessation is effective at reducing much of the harm caused, even after many years of smoking (Pirie 2013; RCP 2018). Smoking cessation interventions are among the most cost‐effective in healthcare, with many estimates suggesting such interventions reduce health service costs overall (Hoogendoorn 2010; NICE 2021; RCP 2018). Many people who smoke would like to stop; however, typically, it takes many attempts to quit before achieving success (Chaiton 2016). This is partly because none of the treatments available to treat tobacco dependence have particularly high success rates and few people use these treatments optimally (Raupach 2014).

### Description of the intervention

Evidence suggests that the most effective way to stop smoking is to use a combination of behavioural and pharmacological support (Hartmann‐Boyce 2019). Five pharmacotherapies for quitting smoking are licensed in at least some parts of the world: nicotine replacement therapy (NRT); bupropion; varenicline; cytisine; and nortriptyline (WHO 2019). E‐cigarettes (EC) are also increasingly used and, in some countries, guidelines support their recommendation by health providers to support a quit attempt (ASH 2021; McNeill 2022; RCP 2018; Warner 2023).Standard Cochrane intervention reviews provide evidence that all these interventions are effective smoking cessation aids (Hajizadeh 2023; Hartmann‐Boyce 2018; Hartmann‐Boyce 2022b; Livingstone‐Banks 2023), and they are all traditionally viewed as competing approaches to smoking cessation, though in some cases they may be used in combination.

### How the intervention might work

NRT is a medication formulated for absorption through the oral mucosa (chewing gum, lozenges, sublingual tablets, inhaler/inhalator, mouth spray, strips), nasal mucosa (spray), or skin (transdermal patches) (Hartmann‐Boyce 2018). Nicotine transdermal patches are worn on the body and deliver a nicotine dose slowly and passively through the skin. Other types of NRT (e.g. gum or lozenge), deliver nicotine faster (described collectively here as fast‐acting NRT).

Nicotine is one of the vehicles of tobacco addiction and neuroadaptations in response to repeated inhalation, which means that when a person stops smoking tobacco, they experience withdrawal symptoms (including urges to smoke and aversive mood and physical symptoms). The aim of NRT is to replace the nicotine that the person smoking would have been receiving, ameliorating withdrawal. Inability to tolerate withdrawal accounts for most cases of early relapse to smoking. After some weeks, the urges to smoke abate, and nicotine can be stopped without precipitating withdrawal symptoms in most people. NRT is available worldwide and the World Health Organization (WHO) deems it an essential medicine; i.e. a medicine that satisfies a priority health need and that people should have access to in sufficient amounts at all times (WHO 2021b).

Varenicline and cytisine are both nicotine receptor partial agonists (Livingstone‐Banks 2023). They activate the nicotinic receptors in the brain, usually activated by nicotine to release dopamine, and prevent nicotine from further activating these receptors. This appears to relieve withdrawal symptoms and reduce the rewarding effects of tobacco smoking. Current evidence suggests that both cytisine and varenicline are efficacious cessation treatments (Livingstone‐Banks 2023), with varenicline having historically been used more extensively worldwide, and deemed an essential medicine by the WHO (WHO 2021b). However, in 2021 Pfizer announced a recall of varenicline because it exceeded acceptable intake limits of a nitrosamine impurity, called N‐nitroso‐varenicline. While this is believed to only be temporary, it has led to worldwide shortages at the time of writing. Cytisine is only available in some countries. However, it is has been identified as a potentially attractive treatment option due to its lower cost relative to other smoking cessation treatments. It is currently undergoing further trials with a view to obtaining licences for worldwide use (Courtney 2021; NCT03709823; Nides 2021).

Bupropion and nortriptyline are antidepressant treatments that have also been used for smoking cessation (Hajizadeh 2023). It is not entirely clear why these two antidepressants can help people to stop smoking. Not all antidepressants are effective cessation aids, suggesting that the mechanism of action is separate from their antidepressant actions. Some antidepressants may have a specific effect on neural pathways or receptors that underlie nicotine addiction. Many countries have licensed bupropion as a smoking cessation aid and it is also deemed an essential medicine by the WHO (WHO 2021b), whereas nortriptyline is licensed for this purpose in New Zealand only.

EC appeared on the market in 2006, and are electronic devices that heat a liquid into an aerosol for inhalation, termed vaping (Hartmann‐Boyce 2022b). The liquid usually comprises propylene glycol and glycerol, with or without nicotine and flavours, and is stored in disposable or refillable cartridges or a reservoir. Although EC are banned in some countries, vaping is currently legal in the UK, the EU, the USA, Canada and New Zealand (among other countries) (Warner 2023). As many EC contain nicotine, they could function as a form of NRT. Indeed, there is evidence that they are effective cessation aids and that they are more effective than traditional NRT (Hartmann‐Boyce 2022b). In some countries, EC are classed as a tobacco product; however, as they do not contain any tobacco constituent apart from nicotine, which is also contained in products that are not classed as tobacco products, we do not consider them as such here.

Due to the number of people who do not manage to quit smoking, or who relapse to smoking despite using these interventions, as well as providing these interventions in isolation there has been an interest in combining them. This could capitalise on the different mechanisms of action to combat tobacco addiction from multiple angles (Ebbert 2010).

### Why it is important to do this review

Against a backdrop of finite resource, and when dealing with a health behaviour so resistant to change, it is particularly important to pinpoint the treatment strategies that work best, focus available efforts and funds on these approaches, and promote them to the general public. This requires data on comparative effectiveness. Additionally, it is important to consider success rates alongside the harms and tolerability of available interventions. Tolerability has an impact on adherence to pharmacological treatment (Balmford 2010; de Dios 2012), which, in turn, affects quitting success (Raupach 2014). Although there is evidence that NRT is a safe and effective medication for smoking cessation (Hartmann‐Boyce 2018), adverse effects such as skin irritation when using patches, or irritation of the nose, throat or eyes when using a nasal spray, can lead smokers to discontinue treatment (Raupach 2014). Equally, a highly efficacious treatment may not be the best approach if it results in serious health problems. Questions remain around the potential harms of some licensed cessation pharmacotherapies; for example, there have been concerns regarding the mental health effects of varenicline and the effects of bupropion on the risk of seizure (Hajizadeh 2023; Moore 2011; Pesola 2002). As EC are a relatively new approach to quitting smoking, the evidence is still accumulating on potential harms and there is substantial debate over their potential effects on health (Hartmann‐Boyce 2022b).

Two network meta‐analyses (NMA) investigating the comparative effectiveness of pharmacological smoking cessation treatments in the general population have previously been carried out; one published in 2013 (Cahill 2013) and the other with searches conducted up to February 2019 (Thomas 2021). The former NMA did not include EC and the latter did not include cytisine or nortriptyline as competing treatment options. This review strengthens the evidence base by including all studies of approved intervention options across the world (i.e. any forms of NRT, EC, varenicline, cytisine, bupropion or nortriptyline), published up to April 2022. We used component network meta‐analysis (cNMA) to explore additional questions beyond comparing different medicine types. CNMA enables us to split our interventions of interest into the constituent components, allowing direct and indirect comparisons between them (Freeman 2018), and investigation of potential effect moderators, such as participants' pre‐existing co‐morbidities and provision of behavioural support.Having more detailed information on how to use and provide smoking cessation medications, and EC to maximise their benefits and tolerability whilst minimising harms, provides more information to help pinpoint the most and least effective interventions. This, in turn, has the potential to reduce healthcare costs, the burden on practitioners and people who smoke, and the burden of disease and death associated with smoking in the general population. These are key drivers of health inequalities and they have a considerable negative impact on individuals, health services and economies.

## Objectives

To investigate the comparative benefits, harms and tolerability of different smoking cessation pharmacotherapies and e‐cigarettes, when used to help people to stop smoking tobacco.

To investigate:

- how the different characteristics of smoking cessation pharmacotherapies and EC interventions (e.g. intervention subtype, dose, length of treatment, whether the intervention is used pre‐quit as well as from quit date or from quit date only) influence benefits, harms and tolerability; and
- whether the existence of participant comorbidities and provision of behavioural support suggest different optimal intervention strategies.

## Methods

### Criteria for considering studies for this review

#### Types of studies

Randomised controlled trials (RCTs) and cluster‐RCTs (cRCTs). Factorial trials and non‐factorial multi‐arm trials were included provided they contained at least one pair of study arms that were balanced with respect to factors other than the interventions of interest. We excluded cross‐over RCTs as it is impossible to assess the effects of a particular smoking cessation intervention on abstinence in the long‐term using these studies. As there are numerous trials of smoking cessation pharmacotherapies, we excluded non‐randomised studies (including quasi‐RCTs). We included studies regardless of language or publication type.

#### Types of participants

Adults (aged 18 years or older) who smoke cigarettes. People using more than one type of tobacco were included as long as cigarette smoking was an inclusion criterion and the trial met all other eligibility criteria. However, we excluded studies that solely recruited pregnant women, as some of the interventions being assessed in this review are unlikely to be offered to this population. Similarly, we excluded young people (under the age of 18 years) as some of the interventions of interest are not available to, or licensed for, young people. Excluding these two populations ensured that all included interventions were jointly randomisable (a key assumption of NMA) (Higgins 2022).

#### Types of interventions

Any pharmacotherapies and technologies approved for tobacco smoking cessation worldwide at the time of writing (i.e. any forms of NRT, EC, varenicline, cytisine, bupropion or nortriptyline), including combination use of more than one of these intervention types. Although some countries allow the use of smokeless or heated tobacco products as harm reduction products, these products were excluded from this review as they are not typically used to quit smoking and they contain tobacco leaf. We included interventions that described themselves as 'relapse prevention' only when they were delivered to people who were still smoking tobacco at study enrolment.

Studies were not eligible if one of the study arms received an additional intervention component whose effects could not be separated from the pharmacotherapy or EC interventions of interest (e.g. where behavioural counselling or a financial incentive was only provided in one study arm). However, studies that provided an additional component (e.g. behavioural support) equally to all included study arms were eligible. We excluded trials that asked participants to reduce the amount they smoked, where complete quitting was not a goal of the study intervention.

Where appropriate, we aimed to evaluate the comparative effects of the following component types:

- intervention type (nicotine, varenicline, nortriptyline, bupropion, cytisine);
- nicotine delivery mode (EC, patch, other fast‐acting methods, e.g. gum or lozenge);
- nicotine dose;
- intended duration of use;
- tapering of nicotine dose;
- timing of intervention (in relation to quit day, i.e. pre‐quit as well as from quit date or from quit date only), and whether any pre‐quit pharmacotherapy is used while reducing to quit or while smoking as usual.

For a full list of components, see Appendix 1.

Relevant comparators included:

- no pharmacotherapy and no EC intervention;
- placebo pharmacotherapy;
- non‐nicotine/placebo EC;
- another eligible intervention type (e.g. a study comparing NRT with varenicline);
- the same intervention type as provided in the intervention arm, but with a varying component or components (e.g. nicotine 21 mg patch versus nicotine 14 mg patch).

#### Types of outcome measures

Studies had to assess smoking abstinence at least six months following baseline to be eligible for inclusion. This is in line with the standard methods of Cochrane Tobacco Addiction Group (TAG).

##### Primary outcomes

Primary measure of benefit:

- long‐term smoking cessation (i.e. for six months or longer). The preferred outcome is biochemically validated continuous or prolonged abstinence at the longest reported time point, including all participants randomised in their original groups.

Primary measure of harm:

- number of participants reporting serious adverse events (SAEs) between baseline and follow‐up, analysed on a complete case basis, as close to the six‐month follow‐up as possible. Alternatively, if interventions extended beyond the six‐month follow‐up then the measure taken closest to intervention end was used. Exact definitions varied between studies, but SAEs are usually defined as events that result in death, are life‐threatening, require or prolong hospitalisation, result in persistent or significant disability or incapacity, or a combination of these. Examples of SAEs are seizures, potentially fatal overdoses, suicide attempts and deaths.

We did not measure non‐serious adverse events (AEs) as these are typically poorly reported, and are most relevant when assessing the tolerability of an intervention. Instead, we assessed tolerability through our secondary outcome (withdrawal due to intervention), described below.

##### Secondary outcomes

Measure of tolerability:

- number of participants who withdrew from the trial due to pharmacological or EC interventions, measured on an intention‐to‐treat basis, as close to the six‐month follow‐up as possible. Alternatively, if interventions extended beyond the six‐month follow‐up then this was measured as close to intervention end as possible.

### Search methods for identification of studies

#### Electronic searches

We identified all listed included, ongoing and excluded studies in the most recent updates of relevant Cochrane Reviews, at the time of the searches. These reviews covered all of the interventions relevant to this review (i.e. varenicline and cytisine (Cahill 2016); NRT (Hartmann‐Boyce 2018; Lindson 2019); bupropion and nortriptyline (Howes 2020); and EC (Hartmann‐Boyce 2021a)). We also updated the searches for each of these reviews by searching Cochrane TAG's specialised register using the search strategies specified in Appendix 2. The register includes any outputs of tobacco‐related RCTs found within the following databases since their inception, and Cochrane TAG's information specialists maintain it with monthly updates. This review includes the results from searches conducted to 29 April 2022:

- Cochrane's Central Register of Controlled trials (CENTRAL, via CRS‐Web; up to Issue 3, 2022);
- MEDLINE Ovid (1945 to 5 April 2022);
- Embase Ovid (1974 to 4 April 2022);
- PsycINFO Ovid (1806 to 4 April 2022);
- US National Library of Medicine's clinicaltrials.gov trial registry, searched via CENTRAL (Issue 3, 2022);
- WHO International Clinical Trials Registry Platform (ICTRP; www.who.int/ictrp/), searched via CENTRAL (Issue 3, 2022).

For further details of the searches used to populate Cochrane TAG's register, see Cochrane TAG's website (tobacco.cochrane.org/resources/cochrane-tag-specialised-register).

For the Cochrane Review of '*Electronic cigarettes for smoking cessation*' (Hartmann‐Boyce 2022b), we additionally searched MEDLINE (1945 to 1 May 2022), Embase (1974 to 1 May 2022) and PsycINFO (1806 to 1 May 2022) via Ovid and CENTRAL via CRS‐Web (Issue 4, 2022), in line with the review search strategy. As this is a living systematic review with monthly searches, searching multiple databases as well as the register increases the chances of finding the most recent studies for review updates. Results of the EC searches up to 1 May 2022 were incorporated.

There were no restrictions on searching other than for EC studies where the literature was only searched from 2004 onwards as EC were not available before that time (Hartmann‐Boyce 2022b).

#### Searching other resources

We contacted investigators of trials where we had insufficient information to make an eligibility judgement or where an ongoing study appeared to be complete, but no published results were available.

### Data collection and analysis

#### Selection of studies

We uploaded the results of our searches of Cochrane TAG's specialised register into Covidence, which removed most duplicate records. We listed the studies found in existing reviews in an online spreadsheet. Two review authors independently screened each reference or study to establish eligibility (AT, AH, JHB, JLB, NL, TRF of the authors of this review, plus EK, SC, AB, KT who were authors on and involved in screening for the updates of relevant reviews: Hajizadeh 2023; Hartmann‐Boyce 2022b; Livingstone‐Banks 2023; Theodoulou 2023). We screened references in two stages, first screening titles and abstracts. For those that appeared to be eligible, or where after discussion within the team eligibility was still unclear, we retrieved full‐text reports. Two review authors then independently screened each full text for eligibility (second stage). Where there were disagreements between authors, a third review author (of those listed above) screened studies. We reported the results of our screening in a PRISMA flow diagram (Page 2021).

#### Data extraction and management

We extracted the following data from each eligible study using an extraction form designed and piloted by the author team.

- **Study characteristics:** relevant references, study registration details, country, funder, author conflicts of interest, design and unit of randomisation; if a cRCT we also extracted the number of clusters allocated to the intervention and comparator, mean cluster size and intracluster correlation coefficient (ICC), where reported.
- **Recruitment:** recruitment method, setting.
- **Participant characteristics:** number randomised, gender, age, number of cigarettes per day, whether participants were recruited based on them having a pre‐existing condition or being hospitalised, motivated to quit (where participants where selected based on their motivation to quit or not).
- **Intervention and comparator details:** pharmacotherapy (or EC) type and subtype (where relevant), dose (defined as standard for the pharmacotherapy used, lower than standard or higher than standard), length of use (defined as standard for the pharmacotherapy used, shorter than standard or extended), method of delivery, initiation of use (i.e. before or on quit day).
- **Common behavioural support/co‐intervention:** type (no support, self‐help only or mixed, or interactive behavioural support).
- **Smoking abstinence outcome:** definition of abstinence, definition of biochemical validation where relevant, number abstinent per arm, follow‐up point, number of participants followed up at this time point.
- **Harm and tolerability outcomes:** follow‐up point, number of participants reporting SAEs in each arm, number of withdrawals due to intervention in each arm, number of participants followed up at this time point.
- **Risk of bias:** information related to any of the risk of bias domains outlined below, information related to any other potential biases identified.

In line with guidance from the *Cochrane Handbook for Systematic Reviews of Interventions* (Higgins 2022), one review author extracted data on study characteristics, methodology and participant characteristics (of AHa; AT; JLB; NL; SZ; AHo; AR; CT). However, two review authors independently extracted component and covariate data, outcome data and information for risk of bias assessments, with any discrepancies discussed between them (of AHa; AT; JLB; NL; SZ; AHo; AR; CT). Where we could not reach a consensus, we discussed the discrepancy more widely within the review author team until the issue was resolved. Where necessary, we contacted study authors for clarifying information.

Where data had already been extracted in duplicate and risk of bias had already been assessed for eligible studies (because they were already included in a relevant Cochrane Review update: Livingstone‐Banks 2023; Hartmann‐Boyce 2018; Hartmann‐Boyce 2022b; Hajizadeh 2023; Theodoulou 2023), we used those data and assessments rather than re‐evaluate these. Where specific domains had not been evaluated for specific reviews, review authors extracted the required data as described in the previous paragraph.

#### Assessment of risk of bias in included studies

We assessed risk of bias for each included study using the Cochrane RoB 1 tool, assessing the following domains:

- sequence generation (selection bias);
- allocation concealment (selection bias);
- blinding (performance and detection bias);
- incomplete outcome data (attrition bias); and
- other sources of bias (where appropriate; if the bias detected in the study report did not appear to be associated with one of the other domains, e.g. selection bias).

For cluster‐RCTs only, we also assessed the following considerations within the 'other sources of bias' domain:

- recruitment bias due to recruitment of participants to clusters after allocation;
- unbalanced baseline characteristics; and
- whether statistical adjustment had been made to the analysis to account for the potential correlation of effects within clusters.

Where apparent, we also considered selection bias under 'other sources of bias'.

We assessed eachdomain as being at low, unclear or high risk of bias, according to guidance on using RoB 1 in the *Cochrane Handbook for Systematic Reviews of Interventions* (Higgins 2011). We also adhered to standard Cochrane TAG methods when assessing blinding and attrition bias (Hartmann‐Boyce 2023).

We assessed blinding as follows.

- Studies that reported a sufficient blinding procedure, as well as who was blinded, were deemed low risk of bias.
- Where sufficient blinding had not been carried out, we judged studies at low risk of bias if smoking status was measured objectively (i.e. biochemical validation).
- We judged studies at high risk of bias if sufficient blinding did not take place and smoking status was measured by self‐report only. In this case, results may have been prone to differential misreport.

We assessed attrition bias as follows.

- We judged studies at low risk of bias when the following conditions were all met: numbers lost to follow‐up at the longest time point were clearly reported for each group (not just overall, unless the overall percentage lost is less than 10%); the overall number of participants lost at the longest time point was not greater than 50%; and the difference in percentage followed up between groups at the longest time point was not greater than 20%. We also considered results at low risk of attrition bias if the authors reported a sensitivity analysis that indicated the overall direction of effect was not sensitive to different imputation methods for loss to follow‐up.
- We judged studies at high risk of bias when the above thresholds were not met, or in the case of cRCTs, where entire clusters were not followed up.
- We judged studies at unclear risk of bias when the number lost to follow‐up in each group was unclear, and authors did not report a sensitivity analysis based on loss to follow‐up.

We gave each study an overall risk of bias, where studies with at least one domain rated at high risk were deemed to be at overall high risk, studies with all domains rated as low risk were given an overall rating of low risk and all the remaining studies were rated as at unclear overall risk of bias.

Working in pairs, eight individuals (five of whom were review authors) independently assessed risk of bias, resolving any discrepancies by discussion (of AHa; AT; JLB; NL; SZ; AHo; AR; CT).

#### Measures of treatment effect

We report pooled results as odds ratios (OR) with 95% credibility intervals (CrIs) as the statistical model described in Data synthesis is conducted on the log‐OR scale. However, we also considered the absolute effect sizes implied by these pooled estimates and report these in our summary of findings tables. Using the posterior distribution of the proportion of participants experiencing events in the control arms, we obtained the mean event rate in the control arms (i.e. for quit rates, the proportion quitting out of those allocated to the 'no intervention' arms). We used this to estimate the likely event rates among those with the component of interest using the component effect sizes.

#### Unit of analysis issues

For cluster‐RCTs we used data adjusted for clustering effects using the ICC or design effect from the study publication, if reported. For studies that did not report sufficient information, we adjusted for clustering using a design effect calculated from the mean cluster size and an assumed ICC of 0.01, a similar value to that reported in other studies in the included reviews (Hajizadeh 2023; Theodoulou 2023).

Factorial RCTs were split and regarded as two or more substudies if this was necessary in order to isolate the effect of the interventions of interest. For example, a 2 x 2 factorial trial that used varenicline and behavioural support as intervention factors would be split into two substudies: one comparing varenicline versus no intervention, and one comparing varenicline + behavioural support versus behavioural support. A 2 x 2 factorial trial that used varenicline and bupropion as intervention factors would be retained as a four‐arm trial.

#### Dealing with missing data

Participants lost to follow‐up were assumed to still be smoking, as is standard in the field (West 2005), and across reviews produced by Cochrane TAG. We noted the proportion of participants for whom the outcome was imputed in this way, and whether there was either high or differential loss to follow‐up. As described above, we used this information in our risk of bias judgements. The assumption that 'missing = smoking' provides conservative absolute quit rates, and makes little difference to the OR unless dropout rates differ substantially between groups.

For our tolerability outcome (withdrawals due to treatment), participants who were not specifically recorded as withdrawn were not deemed to have withdrawn due to treatment. Therefore, we used the number randomised as our denominator.

In contrast, we intended to assess our outcome related to harms (SAE) as complete case, with those lost to follow‐up not included in our analysis where possible. Assuming those lost to follow‐up have not experienced an SAE is not a valid assumption as participants may be lost to follow‐up because they have experienced an SAE. Therefore, this is the most conservative approach to assessing potential harms. However, in most cases the numbers of participants surveyed about SAEs, and the follow‐up point where this was assessed, were not reported, so we used all participants randomised as the denominator in these cases.

#### Assessment of heterogeneity

We conducted a separate cNMA for each of the three specified outcomes (smoking cessation, SAEs and withdrawal due to intervention). We considered whether any of the eligible studies were too clinically heterogeneous to include in the relevant cNMAs without violating the transitivity assumption, but did not exclude any studies from the analysis on this basis. To judge the extent of heterogeneity, prediction intervals for each component effect were estimated assuming a normal distribution with means equal to the component effects and variance equal to the between‐study variance (Riley 2011).

#### Assessment of reporting biases

There is no established way of assessing reporting bias within cNMAs. However, we adapted existing methods for assessing publication bias in standard systematic reviews by generating a funnel plot for each of the pharmacotherapies of interest versus placebo, and overlaying these plots on top of one another, while aligning the reference lines (representing the overall component effect), and considered studies distributed asymmetrically as potential evidence of publication bias. We created a funnel plot for each of the outcomes (smoking cessation, SAEs, withdrawal due to intervention). These funnel plots are limited in that they exclude study arms that combine two pharmacotherapies.

#### Data synthesis

We used Bayesian cNMA and component network meta‐regression (cNMR) random‐effects models, with adjustment for multi‐arm studies, to evaluate the effectiveness of the components identified above versus no pharmacological/e‐cigarette intervention for the smoking cessation outcome and versus no pharmacological/e‐cigarette intervention and placebo and non‐nicotine/placebo e‐cigarette interventions for the SAE and withdrawals due to treatment outcomes. We also compared components to one another (see Types of interventions). We used this to draw conclusions about which components were most strongly associated with smoking cessation, harms and tolerability. We carried out a cNMA for each outcome using WinBUGS and R, through the R2WinBUGS package (Sturtz 2005). As noted previously, we report pooled results as ORs with 95% CrIs and present these findings in forest plots. Models were constructed similarly to those used by Freeman 2018 and adapted to include a binomial likelihood with logit link for binary outcome.

For each cNMA and cNMR model, we ran three Markov chains with different initial values, each with at least 30,000 iterations, discarding the first 15,000 iterations and with the default thinning interval (equal to 3) set by the R2WinBUGS package to compute summary estimates. We used trace plots to evaluate convergence for each chain for all component effects. We used minimally informative (non‐Jeffrey's) prior distributions for the trials' baseline risks (defined as quit rates, SAE rates and withdrawal rates in the relevant control arms), component effects and between‐trial SD parameter (measured on the log‐odds scale). We excluded studies with zero event rates in all arms.

Due to the extensive dataset, and potential complexity of the models required to adequately describe it, we took a sequential approach to model fitting. Whenever two or more arms of a trial shared the same combination of components, we collapsed the arms into a single arm. If after combining the arms, the study was left with just one arm, we excluded this study from the model. Increasing the number of components resulted in the inclusion of a larger number studies and arms. Some studies contributed information to the analysis of some components and not to others, hence there was variability in the number of studies contributing to each analysis. For each outcome we fitted an initial NMA model, only including the intervention type component, and only including study arms where a single pharmacotherapy type was used. A second model was then fitted for each outcome using the mode of delivery in conjunction with intervention type (i.e. for NRT ‐ patch or NRT other/fast‐acting NRT; for placebo ‐ placebo or non‐nicotine EC), and a third model with the same components as the second model, but also including study arms with more than one intervention type. We excluded components where there was insufficient variability (e.g. duration of use). For all other components, we tested their addition one at a time for each outcome. We compared heterogeneity and model fit across cNMA models using the between‐study standard deviation (SD) and deviance information criterion (DIC). We considered a reduction of three or more as meaningful with regards to DIC. Where a component did affect interpretation and any changes did not appear to worsen model fit the component was retained in the final model.

We predicted the effect of nicotine patch and fast‐acting forms of NRT (the latter also referred to as 'nicotine other') by adding the effects of each component on the log scale, as these are commonly recommended and used in combination in clinical practice.

The statistical codes used for the analyses are available on request from the authors.

#### Meta‐regression

We extended the final Bayesian cNMA models to several cNMR models for each outcome and covariate. Studies with unclear or missing information on covariates were excluded from these analyses. The cNMR models included the following covariates (assessed individually) where relevant to the outcome, as specified below:

- participants selected based on pre‐existing condition or hospitalisation (all outcomes);
- length of follow‐up, defined as greater than 6 but less than 12 months or 12 months and over (cessation only; length of follow‐up was poorly reported for the harm and tolerability outcomes);
- behavioural support (not including contact solely to collect outcome data), defined as no behavioural support; self‐help only; interactive behavioural support (cessation and tolerability outcome only; the existence of behavioural support was deemed unlikely to have any impact on potential harms);
- funded by industry, i.e. pharmaceutical, tobacco or independent EC, defined as no funding, funding of the treatment only or funding of the study (cessation and SAE outcomes only; the reporting of withdrawal was deemed unlikely to have been influenced by funding source);
- quit, SAE, withdrawal rates (based on outcome), in control arms, i.e. baseline risk (all outcomes);
- year of publication (cessation only; there is some evidence to suggest that the reported efficacy of smoking cessation pharmacotherapies has reduced over time; however, there is no evidence of a trend over time for SAEs or tolerability).

We had planned to include study level motivation to quit as a covariate, however this was dropped across all cNMAs due to insufficient heterogeneity across the variable, i.e. most studies recruited participants motivated to quit. We assumed a common effect of each covariate on the component effects, except for 'funded by industry', for which we assumed different effects of funding across pharmacological interventions.

We also tested a different effect of the covariates on the component effects for the control arm quit rate and for funding. We plotted the relationship between the control arm quit rate and the component effects in a scatterplot to help interpretation.

#### Sensitivity analysis

We tested whether the findings from our models were sensitive to the exclusion of the following sets of studies/arms:

- those studies that we judged to be at high overall risk of bias; and
- those arms that had a larger contribution to the DIC than expected (i.e. DIC greater than 3). Where the number of excluded arms in a study was greater than the total number of arms minus 1, then the eligible arms of the study were still included. However, if the number of excluded arms in a study was equal to the total number of arms minus 1, then that study would only be left with one eligible arm for analysis, and would therefore be excluded.

We also planned to test if models were sensitive to the exclusion of studies that solely recruited participants who were hospitalised or on the basis of a pre‐existing condition. However, as this covariate did not have any effect on the meta‐regression models for any of the outcomes we deemed this to be unnecessary, i.e. we already have an indication that our outcomes are not influenced by this participant characteristic.

#### Summary of findings and assessment of the certainty of the evidence

There is no agreed best method for evaluating the certainty of cNMA evidence. Therefore, we used the approach previously used in our cNMA of behavioural interventions for smoking cessation (Hartmann‐Boyce 2021b), after consulting with methodological experts, including the Cochrane Editorial and Methods Department. We evaluated certainty for our component effect estimates for our primary outcomes by drawing upon the principles set forward for GRADE evaluations for NMA (Puhan 2014), with adaptations to some domains to better suit cNMA.

We present modified summary of findings tables presenting effect estimates and GRADE evaluations for each component of the primary efficacy outcome final analysis (varenicline; cytisine; nicotine patch; fast‐acting NRT/nicotine other; nicotine EC; non‐nicotine/placebo EC; bupropion; nortriptyline; nicotine tapering), assessed at six months follow‐up or longer, and the primary SAE outcome final analysis (varenicline; cytisine; nicotine patch; fast‐acting NRT/nicotine other; nicotine EC; non‐nicotine/placebo EC; bupropion; nortriptyline) assessed at various time points (often not specified). We used an adapted version of an approach proposed by Yepes‐Nunez 2019. The key principles of this are outlined below.

- **Risk of bias** assessed by evaluating whether the sensitivity analysis removing studies at high risk of bias meaningfully alters the effect estimate.
- **Imprecision** assessed using the CrIs for individual components and the number of events in studies including that component.
- **Inconsistency** assessed by considering the interpretation of prediction intervals, and if they altered interpretation compared to only considering CrI, as well as, where relevant, considering I^2^ values from pairwise meta‐analyses, as per Hartmann‐Boyce 2021b.
- **Indirectness** assessed by considering data from both pairwise comparisons (as per the reviews investigating the individual; pharmacotherapies/EC) and the cNMA, as well as considering the impact of covariates on component effect estimates.
- **Publication bias** assessed using comparison adjusted funnel plots, as described in Assessment of reporting biases.

Judgements were made through discussion between two review authors (NL and JHB).

## Results

### Description of studies

Note: some of the tables below are hosted on an open‐access repository as, due to their size, incorporating them in the main text was not feasible. These are referred to as supplementary tables throughout, annotated as S1, S2, etc., and can be found at: http://dx.doi.org/10.5287/ora-zr7zypv5w.

#### Results of the search

Updated searches from the relevant Cochrane Reviews identified 1944 records. Following removal of duplicates, these in addition to the 962 studies listed as already included, excluded or ongoing in the relevant Cochrane Reviews and one record brought to our attention by an expert in the field, resulted in 2631 records/studies to be screened. We excluded 1337 records at this stage, leaving 1294 for full‐text screening. Seven hundred and thirty‐five full‐text records/studies were then excluded, reasons for which are listed in supplementary table S1. Three hundred and thirty‐two studies were found eligible for this review; however, only 319 provided data suitable for cNMA. The characteristics of these studies are summarised below and further details are available in supplementary table S2. Where a study has been previously included in another Cochrane Review (as noted in the supplementary table S2 'Primary review' column), information can also be found in the 'Characteristics of included studies' tables for the relevant review, and where a study is only included in this review, additional details can be found in supplementary table S3 (for example, 17 studies were identified from the excluded studies lists of relevant reviews). See Figure 1 for more detailed study flow and supplementary table S4 for the included studies reference list.

**1:**
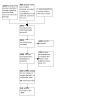
PRISMA flow diagram. *Abbreviations* cNMA: component network meta‐analysis

#### Included studies

Three hundred and thirty‐two RCTs, with approximately 161,100 participants (one study did not report number of participants) met the eligibility criteria for this review. Of these, 319 with 835 study arms and 157,179 participants provided outcome data for at least the efficacy outcome (not all included studies provided data on harms and tolerability). Details of these studies are reported below. The remaining 13 RCTs, with approximately 3921 participants, met the inclusion criteria, but did not provide sufficient data for meta‐analysis. Details of these studies can be found in supplementary table S5.

##### Study types and funding

Of the 319 studies included in analyses, 10 were cluster‐RCTs, 34 were factorial‐RCTs, 1 had mixed parallel and cluster assignment, and the remaining 274 were parallel RCTs. Of the cluster trials, three reported ICCs that we were able to use to make our adjustments in the analyses for our smoking cessation outcome; for six no ICC was reported and we used 0.01 (see Unit of analysis issues); one study claimed that they had adjusted for clustering but the results of their adjustment were consistent with an ICC of zero, so we did not make any adjustment in our analyses. The mixed individual and cluster‐randomised trial did not state how many people were randomised using each method or the number of clusters, so we were unable to make an adjustment.

One hundred and two studies did not receive any funding from the pharmaceutical, e‐cigarette or tobacco industry, 63 received pharmacotherapy or EC from the manufacturers to use in the study, and 118 studies received industry funding. The role of the industry in funding or supplying study materials was unclear in 36 studies.

##### Populations

Eighty‐two of the studies took place in Europe and 166 in the USA and/or Canada. The remainder took place in Australasia, Bangladesh, China, Egypt, Iceland, India, Iran, Israel, Japan, Kyrgyzstan, Pakistan, Republic of Korea, South Africa, South America, Syria, Taiwan, Thailand, Turkey, or across a range of regions. Eighty of the studies recruited participants who had a comorbid condition or were hospitalised. The majority of the studies included in meta‐analyses recruited people who were motivated to quit (234/319), 10 recruited people who were specifically not motivated to quit, 19 did not select participants based on their motivation to quit and 56 did not report on this characteristic.

##### Interventions and comparators

Study arms investigated the following pharmacotherapies/EC interventions in isolation: bupropion (70 arms); cytisine (8 arms); choice of NRT product (24 arms); nicotine EC (16 arms); fast‐acting forms of NRT, i.e. gum, inhalator, lozenge, microtab, mouth spray, nasal spray (107 arms); nicotine patch (122 arms); nortriptyline (8 arms); and varenicline (80 arms).

One hundred and twenty arms tested two active treatment types in combination, as follows: nicotine patch + fast‐acting NRT (i.e. combination NRT; 86 study arms); bupropion + nicotine patch (10 arms); bupropion pill + fast‐acting NRT (8 arms); bupropion + a choice of NRT product (2 arms); nortriptyline + nicotine patch (4 arms); nortriptyline + a choice of NRT product (1 arm); varenicline + bupropion (3 arms); varenicline + nicotine patch (3 arms); nicotine EC + nicotine patch (2 arms); a fast‐acting form of NRT + choice of another NRT product (1 arm).

Two study arms tested bupropion, nicotine patch and fast‐acting NRT in combination. The following were used as comparators: no pharmacotherapy/nicotine EC control (70 arms); placebo EC (8 arms); all other placebos (236 arms).

Twenty‐two of the 319 studies provided no behavioural support as part of their intervention and comparator treatment, 278 provided interactive behavioural support, 13 studies provided self‐help materials only and in 1 study some people received self‐help and some people received interactive support. Whether behavioural support was provided and of what type was not reported in five studies.

##### Outcomes

One hundred and thirty‐one studies measured smoking cessation at six‐month follow‐up, and 164 measured abstinence at 12‐month follow‐up. Thirteen studies measured abstinence at follow‐up points greater than six months but less than 12 months, and the remaining 11 studies at greater than 12 months follow‐up. The longest follow‐up point was five years, which occurred in one study.

One hundred and forty‐six studies reported data on serious adverse events (our harms outcome) and 93 reported data on withdrawals due to treatment (our tolerability outcome). Follow‐up points were not reported consistently for either outcome.

#### Excluded studies

Of the 2630 records/studies from both updated searches and studies listed in relevant reviews, 735 were deemed ineligible for our cNMA at the full‐text screening stage. Primary reasons for exclusion at this stage were inadequate follow‐up (234 records/studies), ineligible study design (117 records/studies), ineligible intervention (97 records/studies), ineligible comparator (71 record/studies), ineligible population (45 record/studies) and ineligible outcomes (records/studies). Further details can be found in supplementary table S1.

#### Ongoing studies

We also identified 53 ongoing studies, details of which can be found in supplementary table S6.

### Risk of bias in included studies

Of the 319 studies included in the meta‐analyses, we judged 51 studies to be at low risk of bias, 104 studies at high risk of bias and the remaining 164 at unclear risk. Risk of bias judgements for individual studies can be found in their parent reviews and in supplementary table S7. Figure 2 summarises risk of bias assessments across domains.

**2:**
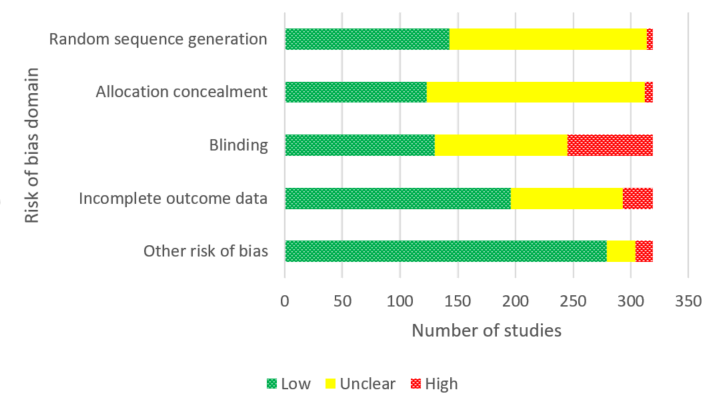
Risk of bias judgements for studies included in the cNMAs by domain *Abbreviations* cNMAs: component network meta‐analyses

#### Allocation

Based on random sequence generation methods and reporting, we deemed 143 studies to be at low risk of bias, 171 at unclear risk and five studies at high risk due to insufficient methods to ensure allocation was truly random. We judged 123 studies to be at low risk of bias with regard to their allocation concealment methods, 189 at unclear risk and seven studies at high risk. In the case of both random sequence generation and allocation concealment, we made unclear judgements where there was insufficient information reported to make a judgement of low or high risk of bias.

#### Blinding

We judged 130 studies to be at low risk of bias based on blinding, 115 to be at unclear risk due to a lack of information and 74 to be at high risk of bias. In the majority of studies that we deemed to be at high risk for this domain, no pharmacotherapy or no EC was used as a comparator rather than a placebo.

#### Incomplete outcome data

We judged 196 studies to be at low risk of attrition bias as follow‐up rates were reported and there was less than 50% loss to follow‐up overall and less than a 20% difference in attrition between arms. We deemed 97 studies to be at unclear risk due to a lack of reporting on attrition, and 26 to be at high risk due to more than 50% attrition, more than 20% difference in attrition between arms, or both.

#### Selective reporting

Selective reporting was assessed as part of the Other potential sources of bias section below.

#### Other potential sources of bias

We deemed 25 studies to be at unclear risk of other bias and 15 to be at high risk, with the remaining 279 studies deemed to be at low risk. This domain took into account any evidence of selective reporting, and for cluster‐RCTs the additional relevant domains described in the Assessment of risk of bias in included studies section above.

### Effects of interventions

See: Table 1; Table 2

#### Smoking cessation

For this outcome, we used 'no pharmacological or e‐cigarette intervention' as the control. See Table 1.

##### Preliminary models

As per our analysis plan (see Data synthesis), we began with an analysis restricted to intervention type (see Appendix 1 for component definitions). This excluded study arms including more than one intervention type, and included 244 trials and 551 study arms with 116,404 participants. All active intervention types showed benefit over placebo and control with CrIs excluding no difference (see supplementary file 8, SD 0.29).

We then ran a model also including arms with more than one intervention type and splitting the nicotine node into nicotine patch; nicotine EC and nicotine other (this includes all fast‐acting forms of NRT other than nicotine EC). The placebo node was also split into placebo EC and all other forms of placebo. This model included 296 trials and 702 study arms, including 142,435 participants. In this model all but one active intervention type showed benefit over placebo and control, with CrIs excluding no difference (supplementary file 8). The one exception was nortriptyline, where the point estimate suggested benefit but the lower bound of the CrI included one. This did not make a difference to the SD (SD 0.29).

In the next set of models we introduced three additional components, one at a time, to test their effects: timing of nicotine treatment (pre‐quit, no pre‐quit); nicotine treatment dose (standard, higher, lower, mixed, missing); nicotine dose tapering (yes, no).

First, we split the nicotine patch and fast‐acting NRT/nicotine other nodes according to timing of treatment (in relation to quit date). This was defined as: pre‐quit treatment use (as well as from quit date) versus treatment use from quit day onwards. This included 301 studies, with 728 arms, including 144,772 participants. Again this did not affect the SD (SD 0.29), and all active interventions had point estimates indicating benefit and CrIs excluding no difference. Comparisons based on timing of nicotine treatment showed no clear evidence of a meaningful difference, and hence we did not include this in the final model (supplementary file 8).

Second, we split the nicotine patch and fast‐acting NRT/nicotine other nodes according to treatment dose. This was defined as: high, low, standard or missing (for classifications of these see Appendix 1). This included 304 studies, with 743 arms, including 145,163 participants. Again this did not affect the SD (SD 0.29), and all active interventions had point estimates indicating benefit and CrIs excluding no difference. As per the previous iteration of the model, comparisons based on dose showed no evidence of a meaningful difference, and hence did not include this in the final model (supplementary file 8).

Finally, we tested splitting the nicotine patch and fast‐acting NRT/nicotine other nodes based on whether or not dose was tapered, defined as yes versus no. This included 299 studies, with 710 arms, in 145,494 participants. There was a slight reduction in SD to 0.28 and all active interventions had point estimates indicating benefit and CrIs excluding no difference. For both types of nicotine node, point estimates for tapering suggested a small but clinically relevant benefit over no tapering, though CrIs overlapped. Due to the magnitude of difference in the point estimates and the consistency of this magnitude across types of nicotine we decided to include nicotine tapering as a component in the final model (supplementary file 8).

We did not enter the component 'intended duration of use' into the model as there was insufficient heterogeneity within intervention types. For the same reason, we only split the nicotine nodes for the components 'timing', 'tapering' and 'dose', as there was insufficient variation in these components for the other intervention types (i.e. varenicline, cytisine, bupropion, nortriptyline, EC). This was true across all outcomes.

##### Final model

Figure 3 shows results from our final cNMA model for our smoking cessation (efficacy) outcome (including the intervention type, nicotine/placebo delivery mode and nicotine tapering components), including all eligible studies. This main analysis included 299 studies and 709 study arms, representing 145,460 participants. There was some variability between studies, which was not fully explained by the components (moderate between‐trial SD 0.28). We chose to present this model (without covariates) as our primary model, as adding in the covariates (as described below) did not substantially improve the SD and did not meaningfully alter component effect estimates. Point estimates for the effect of each component, with 95% CrIs and the numbers of trials, arms and participants contributing data, can be seen in Figure 3. Trace plots indicated good convergence. For some components, CrIs were wide, but for seven interventions, the point estimate suggested clinically important benefit, and the CrI excluded no clinically important difference:

**3:**
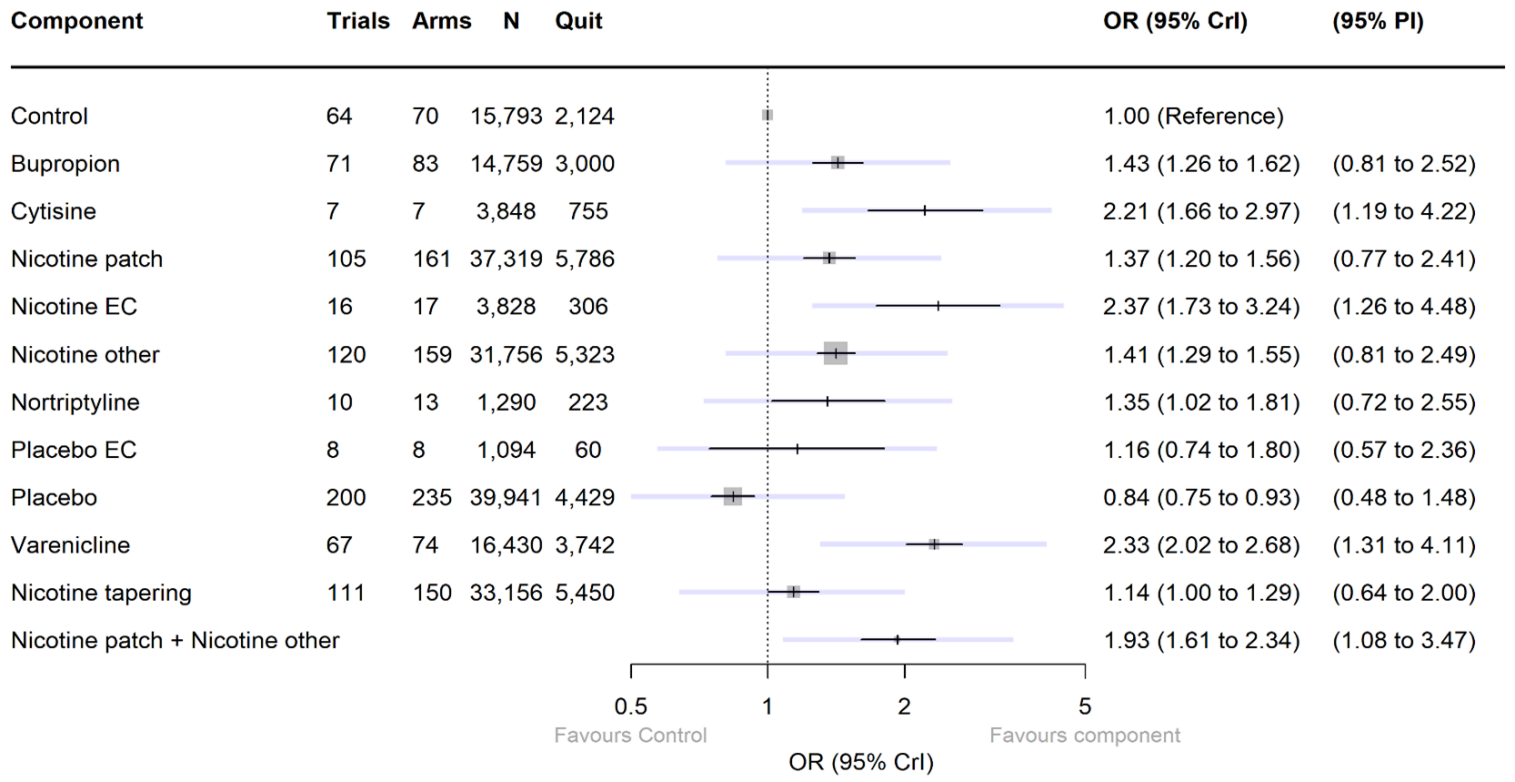
Forest plot illustrating final model for abstinence (efficacy) outcome. Note, darker intervals represent CrI and lighter intervals represent PI. Control: no pharmacological or EC intervention. *Abbreviations* CrI: credibility interval; EC: e‐cigarette; N: number of participants; OR: odds ratio; PI: prediction interval

- varenicline;
- cytisine;
- bupropion;
- nicotine patch;
- fast‐acting NRT (nicotine other);
- combination NRT (nicotine patch plus fast‐acting NRT, calculated as an additive effect);
- nicotine EC.

Of these, varenicline, cytisine, combination NRT and nicotine EC also had prediction intervals excluding the null (Figure 3).

A further two components had point estimates suggesting clinically significant benefit (judged as OR greater than 1.04) and CrIs that included no clinically significant difference, but that excluded clinically significant harm:

- nortriptyline;
- nicotine tapering.

The remaining components (non‐nicotine EC/placebo EC and other placebos) had CrIs where the lower bound included meaningful reductions in the likelihood of achieving abstinence. Non‐nicotine EC/placebo EC had a point estimate indicating a benefit for smoking cessation; however, the lower bound of the CrI also incorporated the possibility of fewer participants quitting when using non‐nicotine EC/placebo EC relative to control.

In head‐to‐head comparisons varenicline, nicotine EC, cytisine and combination NRT all resulted in higher quit rates (with CrIs excluding the null) than bupropion, nortriptyline and single‐form NRT (both patch and fast‐acting NRT). Results indicated varenicline, cytisine, nicotine EC and combination NRT all had similar levels of effectiveness, with point estimates suggested no clinically meaningful difference, but CrIs were wide. For more detail see Appendix 3.

###### Component network meta‐regression

None of the covariates reduced heterogeneity (SD 0.28 to 0.30 for all) when introducing them individually into the final model. However, two covariates did have moderator effects where CrIs excluded the null: selection of participants based on pre‐existing comorbidities or hospitalisation and control arm quit rate. For the former the moderator effect was OR 1.12 (95% CrI 1.03 to 1.23), indicating that the effects of pharmacological interventions, on average, were slightly more pronounced (12% greater) in populations selected for comorbidities and hospitalisations; however, this did not change the clinical interpretation of each of the component effect estimates.

For control arm quit rate (continuous covariate) the OR was 1.05 (95% CrI 1.01 to 1.08), indicating that per every 1% increase in the log odds of quitting, the component effect was 5% greater. In other words, in populations where it is easier to quit, the components have greater effects; conversely, in populations where it is more difficult to quit, the component effects have lower effects. When we assumed that the effect of the control arm quit rate varied by pharmacological intervention, there was some evidence of weaker components effect associated with higher quit rates for nortriptyline, cytisine, nicotine tapering and nicotine EC.

For the remaining covariates we investigated, CrIs for the moderator effects included the null:

- length of follow‐up: OR 1.04, 95% CrI 0.99 to 1.10;
- interactive behavioural support versus no behavioural support: OR 1.03, 95% CrI 0.84 to 1.26;
- self‐help materials versus no behavioural support: OR 1.02, 95% CrI 0.97 to 1.07;
- year of publication: OR 1.00, 95% CrI 1.00 to 1.01; and
- funding of study by industry: OR 0.97, 95% CrI 0.90 to 1.04; when we broke this down by component (by pharmacotherapy type and delivery mode) to investigate interactions all 95% CrIs also included the null.

###### Sensitivity analyses

A prespecified sensitivity analysis excluding studies at high risk of bias (supplementary file 8) did not substantially alter the between‐trials SD (from 0.28 to 0.29). CrIs were wider for all component effects, as would be expected from the reduced sample size; however, all CrIs that previously excluded the null continued to do so. When we removed the 10 study arms with DIC ≥ 3, again it did not substantially alter the between‐trials SD (from 0.28 to 0.27) and had no notable impact on component effect estimates and 95% CrI (supplementary file 8).

#### Serious adverse events

For this outcome, we used 'no pharmacological or e‐cigarette intervention' and 'placebo' as control conditions. See Table 2. Across all study arms, the average proportion of participants experiencing an adverse event was 3%, ranging from 0 to 24%.

##### Preliminary models

As for our smoking cessation outcome (see Data synthesis), we began with an analysis restricted to intervention type, excluding study arms including more than one intervention type. This analysis included 74 trials and 162 study arms, including 49,714 participants. All active intervention types showed evidence of imprecision, with CrIs indicating the potential for a reduced rate of SAEs in the intervention groups versus placebo and control as well as the potential for no difference and an increased rate of SAEs when using the interventions (see supplementary file 9). The SD suggested moderate heterogeneity (SD 0.40).

We then ran a model including arms with more than one intervention type, splitting the nicotine node into nicotine patch, nicotine EC and fast‐acting NRT/nicotine other. This model included 85 trials and 193 study arms, including 56,178 participants. In this model for all intervention types versus control and placebo CrIs included the null (supplementary file 9). The SD for this model reduced very slightly to 0.39.

In the next set of models we introduced dose, tapering and timing, one at a time, as for the previous outcome. When we split the nicotine patch and nicotine other nodes according to timing of treatment the statistical model included 87 studies, with 199 arms, including 57,657 participants. The SD stayed the same (SD 0.39) and comparisons based on timing of treatment showed no evidence of a meaningful difference in the interpretation of effects, hence we did not include this component in the final model (supplementary file 9).

Second, we split the nicotine patch and nicotine other nodes according to treatment dose (high, low, standard or missing; classified in Appendix 1). This included 87 studies, with 199 arms, including 56,742 participants. This resulted in a small increase in the SD (SD 0.41), and continued imprecision across interventions with CrIs including the null in all cases and no difference in interpretation across dose categories. Hence, we did not include this component in the final model (supplementary file 9).

Finally, we tested splitting the nicotine patch and nicotine other nodes based on whether or not dose was tapered (yes or no). This included 86 studies, with 195 arms, in 56,238 participants. There was a slight reduction in SD to 0.38 and again in all cases CrIs incorporated the null. For both types of nicotine, point estimates for tapering suggested a small but clinically relevant benefit over non‐tapering, though CrIs overlapped. Again, we decided not to include this component in the model, as there was no evidence of a difference in the interpretation of the effects dependent on whether tapering occurred or not (supplementary file 9).

##### Final model

Figure 4 shows results from our final cNMA model for our SAE (harms) outcome (including the intervention type and nicotine/placebo delivery mode and excluding the nicotine dosing, timing and tapering components), including all eligible studies. This main analysis included 85 studies and 193 study arms, representing 56,178 participants. There was some variability between studies, which was not fully explained by the components (moderate between‐trial SD 0.39). We chose to present this model (without covariates) as our primary model, as adding in the covariates did not provide sufficient indications of an improvement in between study variation, interpretation or model fit (as described below). Point estimates for the effect of each component, with 95% CrIs and the numbers of trials, arms and participants contributing data, can be seen in Figure 4. Trace plots indicated good convergence. For all components, CrIs incorporated the null. In the cases of bupropion, varenicline and fast‐acting NRT/nicotine other the point estimate favoured no intervention/placebo, and for bupropion and varenicline the lower bound of the CrIs did not include a clinically significant reduction in SAEs. In the cases of cytisine, nicotine patch and nicotine EC, the point estimates favoured the intervention (i.e. SAE rates were lower in the intervention arms); however, the CrIs also incorporated potential harm. For all components, prediction intervals were wide and incorporated benefit, harm and the potential for no difference. Nortriptyline is not represented in this model due to a lack of data (Figure 4).

**4:**
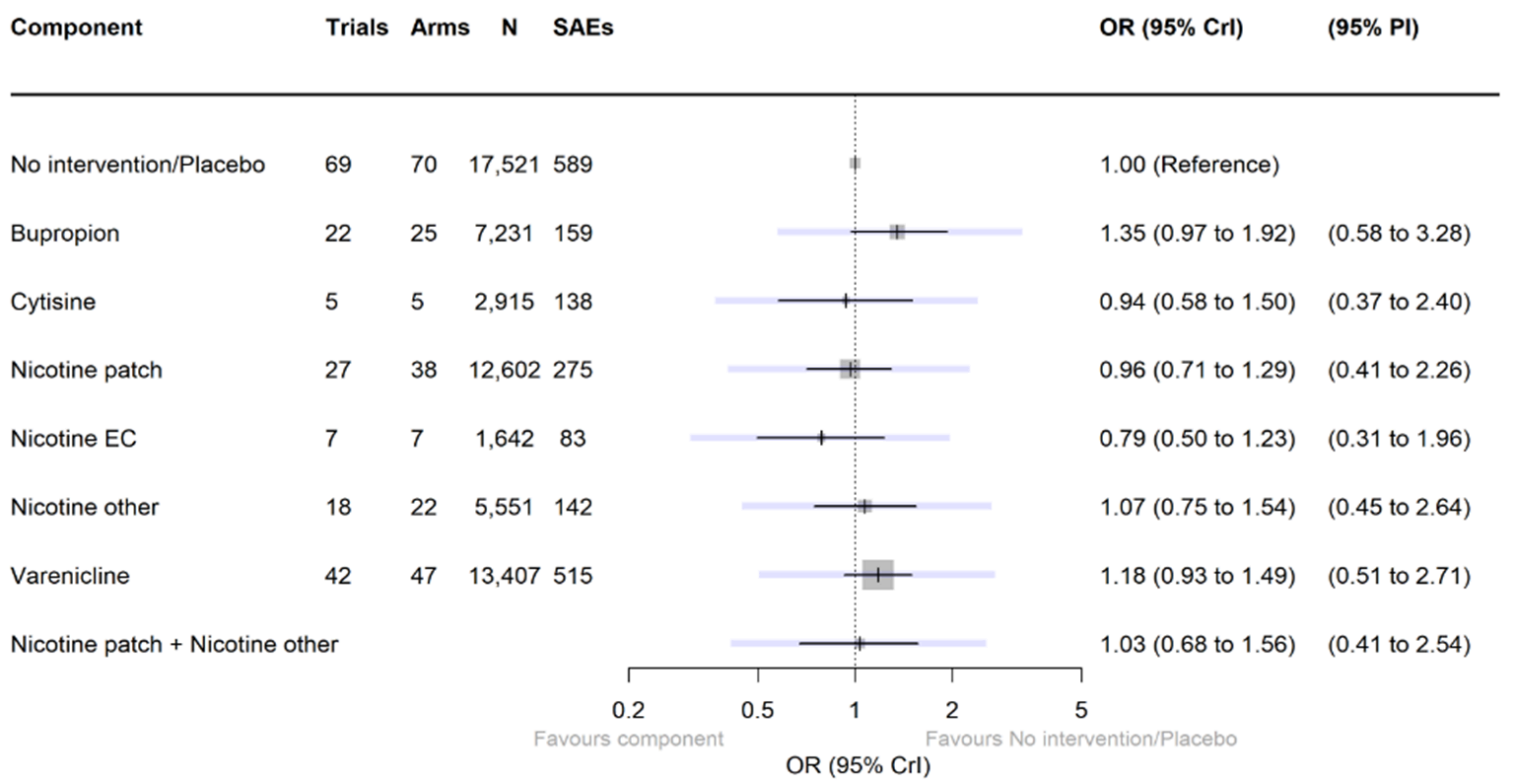
Forest plot illustrating final model for SAE (safety) outcome. Note, darker intervals represent CrI and lighter intervals represent PI. *Abbreviations* CrI: credibility interval; EC: e‐cigarette; N: number of participants; OR: odds ratio; PI: prediction interval

In head‐to‐head component comparisons there was no clear evidence that any components resulted in greater SAE rates than others (Appendix 3).

###### Component network meta‐regression

For the SAE outcome, we deemed only three covariates relevant to the analysis: 1) selection of participants based on pre‐existing co‐morbidities or hospitalisation; 2) control arm SAE rate; 3) funding of study by industry. Neither 'selection of participants' nor 'funding of study by industry' had moderator effects where the CrIs excluded the null, changed the interpretation of component effects or reduced heterogeneity (OR 0.88, 95% CrI 0.64 to 1.22 and OR 1.24, 95% CrI 0.98 to 1.59, respectively). The 'control arm SAE rate' moderator effect did exclude the null (OR 0.83, 95% CrI 0.82 to 0.88) and suggested that component effects were smaller in studies where the control arm had higher rates of SAEs; however, entering it into the model increased the complexity (DIC increased from 906 to 911), hence we decided not to include it in the final model. When including 'control arm SAE rate' in the model, the CrIs for bupropion and varenicline then excluded the null (varenicline: OR 1.67, 95% CrI 1.24 to 2.18; bupropion: OR 1.44, 95% CrI 1.02 to 2.01).

###### Sensitivity analyses

A prespecified sensitivity analysis excluding studies at high risk of bias did not change the interpretation of any of the component effects and increased heterogeneity (median between‐trials SD changed from 0.39 to 0.44). When we removed the two study arms with DIC ≥ 3, it did not substantially alter the median between‐trials SD (from 0.39 to 0.40) and had no notable impact on the interpretation of component effects (see supplementary file 9).

#### Withdrawals due to treatment

For this outcome, we used 'no pharmacological or e‐cigarette intervention' and 'placebo' as control conditions.

##### Preliminary models

We began with an analysis restricted to intervention type, excluding study arms including more than one intervention type. This analysis included 72 trials and 158 study arms, including 44,961 participants. All active intervention types showed greater numbers of withdrawals than control. All but one intervention type (cytisine) had CrIs excluding no difference (see supplementary file 10). The SD suggests moderate heterogeneity (SD 0.36). In the case of cytisine CrIs incorporated the null as well as the possibility of increased withdrawals in the placebo/no intervention control arms.

We then ran a model including arms with more than one intervention type, splitting the nicotine node into nicotine patch, nicotine EC and fast‐acting NRT/nicotine other. This model included 81 trials and 185 study arms, including 48,861 participants. Nicotine EC was not included in the model as so few studies reported data on this outcome. In this model, all interventions types, excluding cytisine and nicotine patch, showed greater numbers of withdrawals in the intervention arms versus control and placebo, with CrIs excluding the null (see supplementary file 10). The CrIs for cytisine and nicotine patch incorporated the null and the potential for increased withdrawals in the active intervention arms; the lower bound of the CrIs for the cytisine component also included the possibility of a clinically meaningful increased rate of withdrawals in the placebo/no intervention arm versus cytisine. The SD for this model increased very slightly to 0.40.

In the next set of models we introduced dose, tapering and timing, one at a time, as for previous outcomes. When we split the nicotine patch and nicotine other nodes according to timing of treatment the statistical model included 82 studies, with 187 arms, including 48,901 participants. This increased the SD slightly (SD 0.39), and some of the CrIs widened to incorporate the null; however, this is not unexpected due to the reduction in participants contributing to each component and there was no clear theoretical basis for why timing may have caused a difference, hence we did not include this component in the final model (see supplementary file 10).

Second, we split the nicotine patch and nicotine other nodes according to treatment dose (high, low, standard or missing; classified in Appendix 1). This included 82 studies, with 190 arms, including 48,921 participants. This resulted in a small increase in the SD (SD 0.40), imprecision across NRT interventions, with CrIs including the null in all cases and no difference in interpretation across dose categories. Hence, we did not include this component in the final model (see supplementary file 10).

Finally, we tested splitting the nicotine patch and nicotine other nodes based on whether or not the nicotine dose was tapered (yes or no). This included 83 studies, with 189 arms, in 49,061 participants. There was a slight increase in SD to 0.40 and, again, in all but one case the CrIs for NRT treatments incorporated the null due to increased imprecision, with the CrIs for tapering overlapping with those for no tapering (see supplementary file 10).

##### Final model

Figure 5 shows results from our final cNMA model for our withdrawals due to treatment (tolerability) outcome (including the intervention type and nicotine/placebo delivery mode and excluding the nicotine dosing, timing and tapering components), including all eligible studies. This main analysis included 81 trials and 185 study arms, representing 48,861 participants. There was some variability between studies, which was not fully explained by the components (moderate between‐trial SD 0.40). We chose to present this model as our primary model, as adding in the covariates did not provide sufficient indications of an improvement in between‐study variation, interpretation or model fit (as described below). As noted above, this model does not include nicotine EC or placebo/non‐nicotine EC because the necessary data were not reported by the relevant studies. Point estimates for the effect of each component, with 95% CrIs and the numbers of trials, arms and participants contributing data, can be seen in Figure 5. Trace plots indicated good convergence. For all components, the point estimate indicated higher withdrawals due to treatment in participants provided with the active treatment, as opposed to placebo/no treatment. For the following five interventions the CrI also excluded no clinically important difference:

**5:**
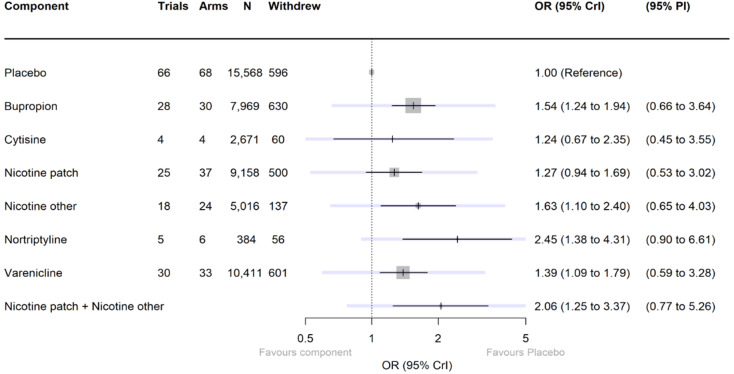
Forest plot illustrating the final model for the withdrawals due to treatment (tolerability) outcome. Note, darker intervals represent CrI and lighter intervals represent PI. *Abbreviations* CrI: credibility interval; EC: e‐cigarette; N: number of participants; OR: odds ratio; PI: prediction interval

- varenicline;
- bupropion;
- fast‐acting NRT/nicotine other;
- combination NRT (nicotine patch and fast‐acting NRT additive effect);
- nortriptyline.

CrIs for nicotine patch included no clinically significant difference, but excluded a clinically significant reduction in withdrawals. Cytisine had CrIs where the lower bound included meaningful increases in the likelihood of withdrawing due to treatment in those receiving placebo/no pharmacological treatment versus active treatment. All of the components had wide prediction intervals incorporating the null as well as potentially increased and decreased withdrawals due to treatment in the intervention arms (Figure 5).

In head‐to‐head comparisons, in all but two cases there was no clear evidence that any components resulted in greater rates of withdrawals than others. When comparing nortriptyline to nicotine patch and combination NRT to nicotine patch, there was some evidence that there were higher rates of withdrawal in the nortriptyline and combination NRT study arms, respectively (Appendix 3).

###### Component network meta‐regression

For the withdrawal (tolerability) outcome we deemed only three covariates relevant to the analysis: 1) selection of participants based on pre‐existing co‐morbidities or hospitalisation; 2) control arm withdrawal rate; 3) interactive behavioural support. None had moderator effects where the CrIs excluded the null, changed the interpretation of component effects, reduced heterogeneity or improved model fit (OR 0.96, 95% CrI 0.69 to 1.36; OR 0.94, 95% CrI 0.86 to 1.04; and OR 0.92, 95% CrI 0.39 to 2.14, respectively). Therefore, we did not include them in our final model.

###### Sensitivity analyses

A prespecified sensitivity analysis excluding studies at high risk of bias did not change the interpretation except for one component effect. The lower bound of the 95% CrIs for fast‐acting NRT/nicotine other shifted toward the null; however, this is to be expected as the data contributing to the analysis reduced and the analysis became more imprecise. Heterogeneity did not decrease meaningfully (median between‐trials SD changed from 0.40 to 0.39). When we removed the three study arms with DIC ≥ 3, again it did not substantially alter the median between‐trials SD (from 0.40 to 0.37) and had no notable impact on the interpretation of component effects (see supplementary file 10).

Narrative descriptions of the findings of the 13 studies deemed to be eligible that did not provide sufficient data for the component network meta‐analysis are available in supplementary table S5. None of the findings appeared to contradict the results that came out of our cNMA.

## Discussion

### Summary of main results

We identified 319 RCTs, with 835 study arms, including 157,179 participants that were eligible and provided sufficient data to include in our component network meta‐analysis (cNMA). We found high‐certainty evidence that nicotine EC, varenicline, cytisine, nicotine patch, fast‐acting NRT/nicotine other and bupropion were associated with higher rates of quitting than no pharmaceutical/EC treatment for quitting smoking. When comparing different smoking cessation pharmacotherapies to one another we found that EC, varenicline and cytisine produced greater quit success than single forms of NRT (i.e. nicotine patches and fast‐acting NRT/nicotine other whether use was tapered or not), bupropion and nortriptyline. However, there was still moderate‐certainty evidence that nortriptyline helped more people to quit smoking than control, and low‐certainty evidence that non‐nicotine/placebo EC helped more people to quit than no pharmacotherapy/EC. We also found evidence that combination NRT (nicotine patches and fast‐acting NRT/nicotine other used together) was more effective than control for smoking cessation, with similar levels of effectiveness to cytisine, varenicline and EC and higher levels of effectiveness than single‐form NRT, bupropion, nortriptyline or non‐nicotine/placebo EC. We did not rate this latter evidence using GRADE as rather than being an individual component, this estimate is the additive effect of two components (both of which are judged to be high certainty).

Evidence on serious adverse events (SAEs) was more limited. More than half of the studies did not report SAE information, and there were insufficient data to include nortriptyline and non‐nicotine/placebo ECs in the models. We found moderate‐certainty evidence that bupropion may cause slightly more SAEs than placebo or no pharmacological treatment, although the CrIs incorporated no difference. There was low‐certainty evidence on nicotine EC, varenicline, cytisine, single‐form NRT and combination NRT; in all cases CrIs incorporated no difference. There was also no clear evidence of a difference for combination NRT (additive rather than component effect). However, when we included the control group SAE rate as a covariate in our model the effect estimates for varenicline and bupropion did exclude the null, indicating more people experienced SAEs when receiving these treatments than when receiving placebo or no pharmacological treatment. Further information could change the interpretation of our component effects and it is important to take into consideration that one of the main reasons for the imprecision in this analysis was the relatively low proportion of participants reporting SAEs.

Data were relatively sparse for our tolerability outcome, i.e. withdrawals due to treatment; however, we did find evidence that varenicline, bupropion, nortriptyline, fast‐acting NRT and combination NRT resulted in more withdrawals due to treatment than placebo or no pharmacological treatment. For nicotine patch and cytisine the point estimates also suggested more withdrawals in the treatment arms, but the effects were imprecise and 95% CrIs also encompassed the potential for no increase relative to placebo/no pharmacological treatment.

We were unable to investigate duration of treatment, as there was insufficient variability in the data across levels of the component, with most studies investigating a 12‐week treatment duration. The remaining components ‐ dose, timing of the intervention (i.e. beginning pre‐quit versus beginning on quit day) and tapering of nicotine treatment ‐ did not affect interpretation of the outcomes, apart from nicotine tapering in the case of abstinence. Low‐certainty evidence indicated that nicotine patch and fast‐acting NRT/nicotine other were more effective when the dose was tapered down toward the end of treatment as opposed to staying on the standard dose and stopping nicotine treatment abruptly.

We did not find any clear evidence that our pre‐specified covariates (i.e. pre‐existing co‐morbidities/hospitalisation, length of follow‐up, provision of behavioural support, industry funding, average control rates or year of publication) moderated the intervention effects we observed, apart from in the case of bupropion and varenicline for our SAE outcome, where including placebo arm SAE rates resulted in the 95% CrIs indicating more SAEs in the intervention arms, excluding the null.

### Overall completeness and applicability of evidence

This review is one of the largest of pharmacotherapies for smoking cessation and we have followed gold standard Cochrane methods to identify eligible studies. We used existing Cochrane Reviews to identify included studies and updated the searches for these reviews up to 29 April 2022. We used a thorough search approach including proactive identification of unpublished data through trial registers and conference abstracts.

As in most research areas, most studies were carried out in higher‐income countries and evidence is lacking in lower‐income countries. Although we would have liked to have looked further at socioeconomic status as a moderator of smoking cessation effects in this review, we decided not to pursue this a priori based on our experience of unsuccessfully trying to extract this information from studies for a similar, previous review (Hartmann‐Boyce 2021b). We hope that future studies will take this into account to allow syntheses incorporating markers of socioeconomic status as a covariate.

A key assumption of network meta‐analysis is the transitivity assumption, i.e. studies should not differ with respect to the distribution of effect modifiers. For this reason we made the decision not to include studies that solely recruited pregnant or young people. Therefore, our findings should not automatically be extrapolated to these populations.

Cytisine is currently not licensed worldwide and nortriptyline is only licensed in New Zealand. NRT, bupropion and varenicline are all currently on the WHO's list of essential medicines; however, at the time of writing there are problems with the availability and supply of varenicline in many parts of the world due to issues with its manufacturing process. Nicotine EC are also banned in some countries. In the RCTs studied here, in most cases, the treatments were provided directly to participants by researchers; however, in reality, this is not the case and even in a setting where a number of different treatments are available, people may find it easier to access some than others, for example, buying treatments like EC or NRT in a supermarket as opposed to accessing varenicline or bupropion through a prescription. Thus, access to treatment may play a role in relapse to smoking even where initial quit attempts are successful and for this reason the effectiveness demonstrated here may not always reflect what we see in the real world.

In addition, it was outside of the scope of this review to investigate all relevant combinations of pharmacotherapies and EC; however, we hope to investigate this further in future. Very few studies investigating EC provided data on tolerability. This is because EC are typically not permitted to be tested as pharmaceutical interventions in trial contexts.

### Quality of the evidence

Of the 319 studies included in meta‐analyses, we judged 51 to be at low risk of bias, 104 studies at high risk and the remaining 164 at unclear risk of bias. Removing studies at high risk of bias did not lead to any meaningful differences in the interpretation of component effect estimates.

For effectiveness, we found high‐certainty evidence that varenicline, nicotine EC, cytisine, nicotine patch, fast‐acting NRT/nicotine other and bupropion all increased quit rates at six months or longer compared to control (Table 1). We judged the evidence showing benefit for nortriptyline to be of moderate certainty, due to imprecision (as the CrI encompassed significant benefit as well as no clinically significant difference). We judged the evidence of a small benefit of non‐nicotine EC/placebo EC to be low certainty due to substantial imprecision (with the CrI incorporating clinically significant benefit as well as harm). We also judged the evidence of benefit for nicotine tapering to be of low certainty: the CrI incorporated no difference as well as a clinically significant benefit and the prediction intervals altered the interpretation of the effect (downgraded one level for imprecision and one level for inconsistency).

For our analyses of harms there were two components (nortriptyline and non‐nicotine/placebo EC) that did not have sufficient evidence to include. For the remaining components, with one exception, we judged the evidence to be of low certainty (Table 2). For varenicline, cytisine, nicotine patch, fast‐acting NRT/nicotine other and nicotine EC we downgraded two levels due to imprecision, as the CrIs were very imprecise. However, we deemed the SAE evidence for bupropion to be of moderate certainty; we downgraded once for imprecision as the CrIs encompassed clinically significant harms as well as the null. Interpretation of the findings related to harms is limited by the fact that studies differed in what they considered to constitute SAEs or whether they could be directly attributed to product use. Studies often did not report this information clearly. This led to high heterogeneity in reported absolute SAE rates between individual studies and between groups of studies of particular intervention types. This variation in reporting also made it unfeasible to analyse the frequency of specific adverse events across the network.

We did not downgrade any of our components based on risk of bias, publication bias or indirectness.

### Potential biases in the review process

This review was carried out according to Cochrane methods, which are rigorous and considered best practice. However, it is impossible to eradicate the possibility of all potential biases. For example, there may have been differences in the ways review authors assessed risk of bias across the studies eligible in previous reviews. The Cochrane Tobacco Addiction Group have made an attempt to minimise this by generating guidelines for assessing risk of bias in RCTs of smoking cessation interventions that is shared with authors of our reviews (Hartmann‐Boyce 2023).

Additionally, some of the authors of this review are authors of included studies; they were not involved in screening, data extraction or risk of bias assessments for their own studies to attempt to mitigate any impact of this.

As highlighted in our previous cNMA (Hartmann‐Boyce 2021b), the cNMA methods used here remain relatively new. We are not aware of any established or agreed way to judge certainty, present summary of findings tables or evaluate publication bias within cNMA. We therefore consulted with the methodological experts within Cochrane and made informed decisions about our approach to each of these areas; other methods may emerge over time. In addition, when estimating the effect of combination NRT, we did so by looking at the additive effect of nicotine patch alone and a fast‐acting form of NRT. We did not assume an interaction and so the prediction is purely the sum of the individual component effects (on a log scale). It is possible that the effect of one component could be lowered by the presence of another effective component. We have not tested the potential effects of delivering more than one pharmacotherapy for other combinations of treatment but hope to pursue this in a separate publication.

Considering potential publication bias, we took into account the findings of studies that we deemed to be eligible but that did not provide enough data to be included in our cNMA (see supplementary file 6), and generated funnel plots for each of our three outcomes. In terms of the former, there was no indication that study findings contradicted the findings reported here. Where it was suspected that studies may have found no evidence of an effect, these studies were small and so would not be expected to change the interpretation of our findings. Our funnel plot for abstinence (Appendix 4) showed that there may be some evidence of asymmetry for varenicline, i.e. a lack of smaller studies showing lack of efficacy; however, due to the amount of evidence in this area showing beneficial effects, additional small studies favouring control would not be expected to change the interpretation of our results. Our funnel plots for our SAE and withdrawals outcomes showed no clear evidence of asymmetry (Appendix 4). There is no established way to assess reporting bias or create funnel plots for cNMA; our method is, to the best of our knowledge, the first time this has been done, and due to the nature of its design, it excludes study arms providing combinations of pharmacotherapies.

### Agreements and disagreements with other studies or reviews

As reflected in the ratings of the certainty of the evidence presented here, the findings of this review are comparable with the findings of all the relevant Cochrane Reviews, which conducted pairwise meta‐analyses investigating the same treatments (Hajizadeh 2023; Hartmann‐Boyce 2018; Hartmann‐Boyce 2022b; Livingstone‐Banks 2023; Theodoulou 2023). Two previous reviews used similar methods; both conducting NMA rather than cNMA. The first was the precursor to this review (Cahill 2013). Cahill 2013 did not include EC, but for the other treatments found effects similar to those reported here, i.e. varenicline was found to be superior to any single type of NRT (patch or fast‐acting) and to bupropion. Neither patch nor fast‐acting NRT were found to be more effective than one another, and combination NRT outperformed single formulations and was potentially as effective as varenicline. There was also some evidence that cytisine and nortriptyline were more effective than placebo. A more recent network meta‐analysis (searches up to February 2019) investigated the effects of varenicline, bupropion, NRT and EC, not including cytisine and nortriptyline trials (Thomas 2021). They concluded that the treatments all showed favourable effects on quitting compared with placebo. Varenicline showed the most pronounced positive effects, with slightly lower, comparable effects found for NRT and bupropion. EC also showed the potential for increasing quit rates but the estimates were extremely imprecise. Thomas 2021, in addition, found very imprecise effects for SAE data and raised issues with the reporting of these data. For all treatments, excluding bupropion, they found no clear association with increased SAEs, whereas for bupropion the 95% CrI did exclude the null. Whereas the Thomas 2021 review included 363 studies, we found 319 relevant studies despite our searches being later and our review covering more treatments. We believe the reason for this is that we did not include studies where behavioural support was not matched between arms in order to reduce confounding. Thomas 2021 did include these studies but controlled for them in a sensitivity analysis. These different approaches resulted in similar findings. However, Thomas 2021 concludes that "... this study strengthens the evidence base for the use of varenicline and NRT monotherapies as first‐line choices for tobacco cessation". Our findings suggest that single‐form NRT should not be favoured over nicotine EC, cytisine or combination NRT, where these options are available.

## Authors' conclusions

**Implications for practice**

- We found high‐certainty evidence suggesting that nicotine e‐cigarettes (EC), varenicline and cytisine were associated with the greatest chances of quitting tobacco smoking at six months or longer, with no clear evidence of a difference in effectiveness between the three. The point estimate was slightly lower for combined nicotine replacement therapy (NRT) but credibility intervals (CrIs) overlapped.
- Bupropion (high certainty) and single forms of NRT (nicotine patch and fast‐acting NRT; high certainty) also had point estimates and CrIs indicating benefit. Point estimates for nortriptyline (moderate certainty) and non‐nicotine/placebo EC (low certainty) also indicated possible benefit but CrIs were wide, incorporating no difference and in the latter case, possible harm.
- There is low‐certainty evidence suggesting that gradually reducing the nicotine dose of nicotine‐based treatments when approaching the end of use may result in slightly more favourable quit rates to stopping nicotine treatment abruptly, but CrIs incorporated no difference.
- There was no clear evidence that nicotine dose or timing of nicotine treatment initiation (i.e. pre‐ or post‐quit) had an effect on quit, serious adverse events (SAE) or withdrawal rates. We were unable to assess the effects of treatment length.
- There is moderate‐certainty evidence that bupropion may slightly increase the likelihood of SAEs when compared to no pharmacotherapy/placebo, although there is also a possibility of no increase.
- There is low‐certainty evidence of no clear difference in SAEs for nicotine EC, varenicline, cytisine and single‐form NRT (nicotine patch and fast‐acting NRT) when compared to no pharmacotherapy/placebo. There was also no clear evidence of a difference for combination NRT.
- There were insufficient SAE data to include nortriptyline and non‐nicotine EC in our analysis of potential harms.
- For tolerability, all of the point estimates (for varenicline, cytisine, bupropion, nortriptyline, nicotine patch, fast‐acting NRT and combination NRT) indicated greater dropouts due to treatment in the intervention arms; however, for cytisine and nicotine patch the CrIs also incorporated the null. There were insufficient data to include nicotine EC in this analysis.

**Implications for research**

- Thorough collection and reporting of serious adverse event data is needed across all intervention types to strengthen our certainty in our results for this outcome.
- Direct comparisons between varenicline, cytisine, nicotine EC and combination NRT, particularly focused on harms and tolerability, are needed. These appear to be the treatments associated with the highest quit rates and more direct data on these outcomes would aid clinical decision‐making.
- Further studies should be funded and conducted to investigate these treatments compared to one another in lower‐income countries, in order to improve the geographical and cultural generalisability of the results.
- Based on the scarcity of socioeconomic data reported in the studies included in our previous component network meta‐analysis of behavioural interventions for smoking cessation (Hartmann‐Boyce 2021b), we decided not to attempt to collect these data for this review. However, future studies should collect and report this information, so that it is possible to carry out investigations into socioeconomic status as a moderator of treatment effects.
- Future research should look into optimum combinations of pharmacotherapies and of combination behavioural and pharmacological support for smoking cessation.

## History

Protocol first published: Issue 3, 2022

## Figures and Tables

**6:**
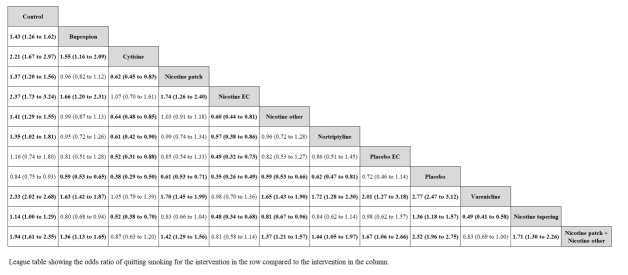
Figures shown in each cell are the odds ratios alongside their 95% credibility intervals. Control: no pharmacological or EC intervention *Abbreviations* EC: e‐cigarette

**7:**
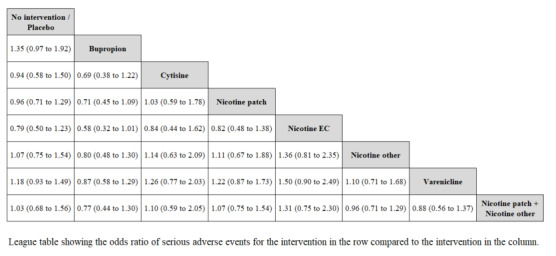
Figures shown in each cell are the odds ratios alongside their 95% credibility intervals. *Abbreviations* EC: e‐cigarette

**8:**
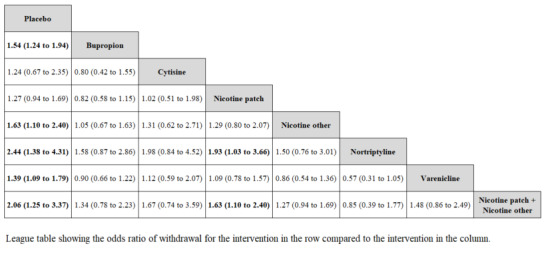
Figures shown in each cell are the odds ratios alongside their 95% credibility intervals. Control: no pharmacological or e‐cigarette intervention

**9:**
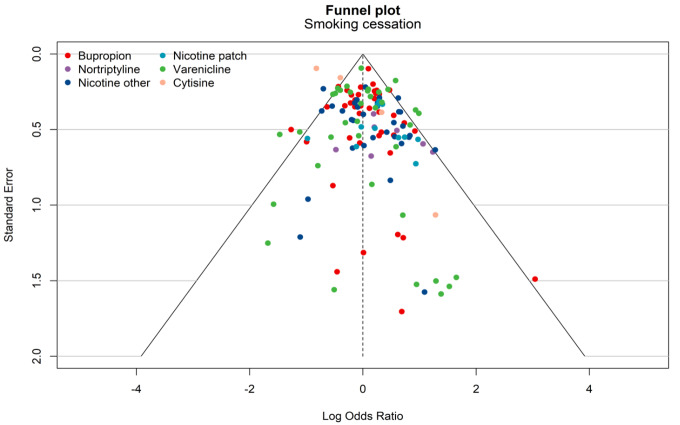
Funnel plot for smoking cessation outcome. Note, the funnel plot is centred at the estimated mean effect for that group, meaning a log(odds ratio) of 0 corresponds to the mean effect for each intervention group (this allows all funnel plots within the group to be superimposed, aiding a holistic assessment).

**10:**
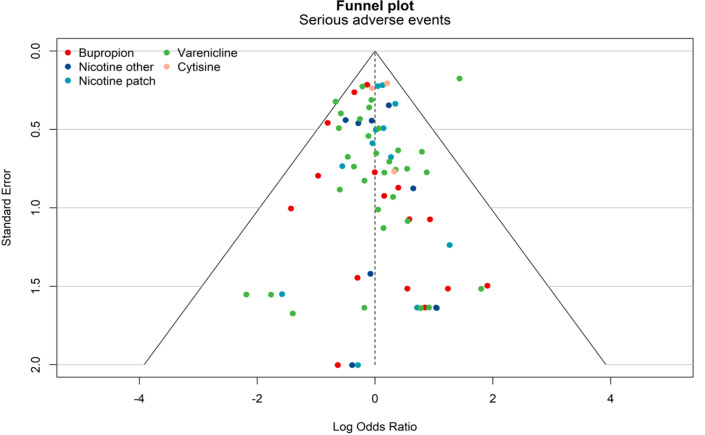
Funnel plot for serious adverse events outcome. Note, the funnel plot is centred at the estimated mean effect for that group, meaning a log(odds ratio) of 0 corresponds to the mean effect for each intervention group (this allows all funnel plots within the group to be superimposed, aiding a holistic assessment).

**11:**
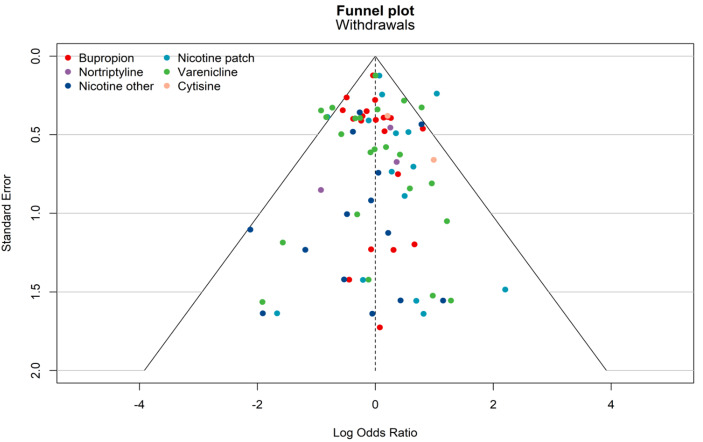
Funnel plot for withdrawals due to treatment outcome. Note, the funnel plot is centred at the estimated mean effect for that group, meaning a log(odds ratio) of 0 corresponds to the mean effect for each intervention group (this allows all funnel plots within the group to be superimposed, aiding a holistic assessment).

## Appendices

### Appendix 1. List of components

#### Intervention type

- Nicotine
- Varenicline
- Cytisine
- Bupropion
- Nortriptyline
- Placebo

#### Delivery mode

- Tablet
- E‐cigarette
- Patch
- Fast‐acting nicotine replacement therapy ('nicotine other')

#### Dose

- Lower than standard
- Standard (nicotine patch: 21/25 milligrams (mg); nicotine gum: 4 mg; nicotine lozenge: 2 mg; nicotine nasal spray: 10 mg; nicotine inhalator: 15 mg/millilitre (mL); nicotine microtab: 2 mg; nicotine mouth spray: 1 mg; nicotine oral strips: 2.5 mg; varenicline 2 mg per day; cytisine: 1.5 mg/tablet 25‐day downward titration schedule; bupropion: 300 mg per day; nortriptyline: 75 mg to 100 mg; EC: 10 mg/mL to 20 mg/mL)
- Higher than standard
- Not applicable (placebo)

#### Intended duration of use

- Shorter than standard (less than 12 weeks)
- Standard (12 weeks)
- Extended (greater than 12 weeks)

#### Tapering

- Yes
- No

#### Timing of intervention (in relation to quit date)

- Pre‐quit, while reducing smoking (as well as from quit date)
- Pre‐quit, without smoking reduction (as well as from quit date)
- From quit day only

### Appendix 2. Search strategies of included reviews

The following search strategies are for the Cochrane Tobacco Addiction Group's specialised register. The 'Last searched' date indicates the date of search for the previous version of the published review. However, for this component network meta‐analysis, the search for each review using the same strategies was updated on 29 April 2022.

**Nicotine replacement therapy versus control for smoking cessation (**Hartmann‐Boyce 2018**)**

*Last searched: 6 July 2017*

#1 NRT: TI,AB,KY,XKY,MH,EMT

#2 (nicotine NEAR2 patch*):TI,AB,KY,XKY,MH,EMT

#3 (nicotine NEAR2 gum):TI,AB,KY,XKY,MH,EMT

#4 (nicotine NEAR2 nasal spray):TI,AB,KY,XKY,MH,EMT

#5 (nicotine NEAR2 lozenge*):TI,AB,KY,XKY,MH,EMT

#6 (nicotine NEAR2 tablet*):TI,AB,KY,XKY,MH,EMT

#7 (nicotine NEAR2 sublingual):TI,AB,KY,XKY,MH,EMT

#8 (nicotine NEAR2 inhal*):TI,AB,KY,XKY,MH,EMT

#9 (nicotine NEAR2 replacement):TI,AB,KY,XKY,MH,EMT

#10 (nicotine NEAR3 therap*):TI,AB,KY,XKY,MH,EMT

#11 #1 OR #2 OR #3 OR #4 OR #5 OR #6 OR #7 OR #8 OR #9 OR #10

**Different doses, durations and modes of delivery of nicotine replacement therapy for smoking cessation (**Lindson 2019**)**

*Last searched: 30 April 2018*

#1 NRT: TI,AB,KY,XKY,MH,EMT

#2 (nicotine NEAR2 patch*):TI,AB,KY,XKY,MH,EMT

#3 (nicotine NEAR2 gum):TI,AB,KY,XKY,MH,EMT

#4 (nicotine NEAR2 spray*):TI,AB,KY,XKY,MH,EMT

#5 (nicotine NEAR2 lozenge*):TI,AB,KY,XKY,MH,EMT

#6 (nicotine NEAR2 tablet*):TI,AB,KY,XKY,MH,EMT

#7 (nicotine NEAR2 sublingual):TI,AB,KY,XKY,MH,EMT

#8 (nicotine NEAR2 inhal*):TI,AB,KY,XKY,MH,EMT

#9 (nicotine NEAR2 strip*):TI,AB,KY,XKY,MH,EMT

#10 (nicotine NEAR2 microtab*):TI,AB,KY,XKY,MH,EMT

#11 (nicotine NEAR2 replacement):TI,AB,KY,XKY,MH,EMT

#12 (nicotine NEAR3 therap*):TI,AB,KY,XKY,MH,EMT

#13 #1 OR #2 OR #3 OR #4 OR #5 OR #6 OR #7 OR #8 OR #9 OR #10 OR 11 OR 12

**Antidepressants for smoking cessation (**Howes 2020**)**

*Last searched: 10 May 2019*

#1 (bupropion or zyban):TI,AB,MH,EMT,KY,XKY

#2 nortriptyline:TI,AB,MH,EMT,KY,XKY

#3 (monoamine oxidase inhib*):TI,AB,MH,EMT,KY,XKY

#4 (moclobemide or selegiline or lazabemide):TI,AB,MH,EMT,KY,XKY

#5 (SSRI* or ((selective serotonin re‐uptake inhibitor*) or (selective serotonin reuptake inhibitor*))):TI,AB,MH,EMT,KY,XKY

#6 (fluoxetine or sertraline or paroxetine or zimelidine):TI,AB,MH,EMT,KY,XKY

#7 (doxepin or imipramine or tryptophan or venlafaxine):TI,AB,MH,EMT,KY,XKY

#8 ((john* wort) or hypericum):TI,AB,MH,EMT,KY,XKY

#9 #1 OR #2 OR #3 OR #4 OR #5 OR #6 OR #7 OR #8

**Nicotine receptor partial agonists for smoking cessation (**Cahill 2016**)**

*Last searched: 12 May 2015*

(cytisine or Tabex or dianicline or varenicline or Champix or Chantix):TI,AB,MH,EMT,XKY,KY,KW

MeSH DESCRIPTOR Nicotine WITH AG AI

MeSH DESCRIPTOR Nicotinic Agonists

MeSH DESCRIPTOR Nicotinic Antagonists

nicotinic agonist*:TI,AB,MH,EMT,XKY,KY,KW

nicotinic antagonist*:TI,AB,MH,EMT,XKY,KY,KW

nicotin* NEAR2 partial:TI,AB,MH,EMT,XKY,KY,KW

#1 OR #2 OR #3 OR #4 OR #5 OR #6 OR #7

**Electronic cigarettes for smoking cessation (**Hartmann‐Boyce 2022b**)**

*Last searched: 1 October 2021*

#1 exp case control studies/ or exp cohort studies/ or Case control.tw. or (cohort adj (study or studies)).tw. or Cohort analy$.tw. or (Follow up adj (study or studies)).tw. or (observational adj (study or studies)).tw. or Longitudinal.tw.

#2 (e‐cig* or ecig* or electr* cigar* or electronic nicotine).mp. or (vape or vapes or vaporizer or vapourizer or vaporiser or vapouriser or vaper or vapers or vaping).ti,ab. or exp Electronic Nicotine Delivery Systems/

#3 (randomized controlled trial or controlled clinical trial).pt. or randomized.ab. or placebo.ab. or clinical trials as topic.sh. or randomly.ab. or trial.ti.

#4 exp animals/ not humans.sh.

#5 3 not 4

#6. 2 and 5

#7 1 and 2

#8 6 or 7

#9 smoking cessation.mp. or exp Smoking Cessation/

#10 tobacco cessation.mp. or "Tobacco‐Use‐Cessation"/

#11 (nicotine dependence or tobacco dependence).mp.

#12 exp Smoking/th

#13 "Tobacco‐Use‐Disorder"/

#14 Smoking reduction/ or Smoking reduction.mp.

#15 exp Pipe smoking/ or exp Tobacco smoking/ or exp Tobacco Products/

#16 ((quit* or stop* or ceas* or giv* or abstain* or abstinen*) adj5 (smoking or smoke* or tobacco)).ti,ab.

#17 exp Tobacco/ or exp Nicotine/

#18 9 or 10 or 11 or 12 or 13 or 14 or 15 or 16 or 17

#19 8 and 18

### Appendix 3. League tables

Smoking cessation outcome: Figure 6; Harms outcome: Figure 7; Withdrawal due to treatment outcome: Figure 8

### Appendix 4. Funnel plots

Smoking cessation outcome: Figure 9; Harms outcome: Figure 10; Withdrawals due to treatment outcome: Figure 11

## Differences between protocol and review

- In the protocol we specified that we would look at the effects of, and interactions between, types of intervention used in combination. For reasons of pragmatism we only look at the additive effect of combining nicotine patch and fast‐acting nicotine replacement therapy (NRT) in this paper (as that is the most commonly used and recommended combination). We did not investigate any interaction. However, we plan to further examine other interventions used, or that have the potential to be used, together in clinical practice in a secondary paper.
- We give further detail regarding the eligibility of factorial and cross‐over trials in this review. This is not based on any change from protocol but rather the specification of more detail in this iteration.
- We used placebo/non‐nicotine e‐cigarette (EC) combined with non‐pharmacotherapy/EC control as the reference group for the SAE outcome as well as the tolerability outcome. Serious adverse events (SAEs) were sparsely reported in studies that had a non‐pharmacological/EC control. This is understandable as it is impossible that SAEs in the control arm could be linked to the intervention. Therefore, placebo/non‐nicotine EC was deemed a more appropriate comparator, and was prespecified as such for the tolerability outcome.
- We made the following changes regarding data extraction: 1) we did not extract all eligibility criteria for each study as this was deemed unfeasible; 2) we did not extract the proportion pregnant, behavioural support ‐ mode of delivery, behavioural support ‐ overall duration of support, as pre‐extraction it was decided that these data would take too long to collect whilst not being directly used in analyses; 3) rather than extract the proportion of people recruited with pre‐existing conditions or hospitalised we noted where participants were recruited on the basis of them having a pre‐existing condition or being hospitalised.
- In this review, we provide more detail on how we dealt with factorial trials in the Unit of analysis issues section. This is not based on any change from protocol but rather the specification of more detail in this iteration.
- We collapsed a number of components due to collinearity, e.g. all varenicline provided in pill form, post quit date with lack of tapering. This led to simplified models.
- We collapsed some other components to increase power where there was a lack of data in some categories. For example, for the placebo component we grouped together placebo (i.e. non‐nicotine) EC and all other placebos. For the delivery mode we grouped all forms of fast‐acting NRT (e.g. gum, lozenge, microtab etc.) into one fast‐acting NRT/nicotine other category.
- We did not carry out planned sensitivity analyses for people with co‐morbidities or who were hospitalised as planned. This is because our meta‐regression models did not find any meaningful impact of adding this covariate on component effects and so sensitivity analyses would have similarly found no meaningful effect.
- We added prediction intervals to the final models for each outcome to quantify the impact of between‐study heterogeneity and use this in our grading of the certainty of the evidence.
